# Ferroptosis inhibition as a renoprotective strategy in cisplatin-induced acute kidney injury: multilevel meta-analysis of mechanistic biomarkers

**DOI:** 10.3389/fmed.2026.1801504

**Published:** 2026-05-04

**Authors:** Burcu Yuksel, Nurullah Eryilmaz

**Affiliations:** 1 Kocaeli University, İzmit, Türkiye; 2 University of Bath, Bath, United Kingdom

**Keywords:** acute kidney injury, cisplatin, ferroptosis, glutathione, GPX4, meta-analysis, renoprotection

## Abstract

**Background:**

Cisplatin-induced acute kidney injury (CI-AKI) is a severe, dose-limiting nephrotoxicity affecting up to 30% of patients. Emerging evidence highlights ferroptosis—an iron-dependent form of regulated cell death—as a key mechanism in cisplatin-induced tubular damage. However, the overall effectiveness of various ferroptosis inhibitors remains unclear. This study aims to synthesise preclinical evidence to assess the renoprotective potential of natural and synthetic agents targeting the ferroptotic cascade.

**Methods:**

Following PRISMA 2020 guidelines, a systematic review and multilevel meta-analysis were conducted across PubMed and Web of Science. From 58 unique preclinical studies (265 effect sizes), we extracted data on renal function (BUN, SCr) and ferroptotic biomarkers (GPX4, GSH, MDA, 4-HNE, Fe^2+^). Standardised mean differences (Hedges’ g) were pooled using a three-level random-effects model, and moderators were assessed using multilevel meta-regression.

**Results:**

Both natural and synthetic interventions significantly alleviated renal dysfunction, markedly reducing BUN (Intercepts *β* = −3.84 and −4.06, respectively; *p* < 0.001) and serum creatinine. Outcome type emerged as a dominant moderator (*Q_M_* > 500), with the most pronounced therapeutic shifts observed in the restoration of the antioxidant shield: GPX4 (*β* = 8.56 for natural, 8.71 for synthetic) and GSH (*β* = 6.99 for natural, 6.33 for synthetic) (*p* > 0.05). Significant residual heterogeneity was observed (*Q_E_*, *p* < 0.001), and PET–PEESE analyses indicated small-study effects, suggesting that the magnitude of the pooled estimates may be inflated. Although the Trim and Fill adjustment did not alter the direction of findings, the overall results should be interpreted with caution.

**Conclusion:**

Inhibition of ferroptosis is a promising preclinical strategy to mitigate CI-AKI, with restoration of the System Xc−/GSH/GPX4 axis emerging as the primary mechanistic driver of renoprotection. These results provide a quantitative mechanistic consensus supporting further investigation and clinical development of ferroptosis-targeted adjuvants to widen the therapeutic window of cisplatin-based chemotherapy.

## 1 Introduction

Cisplatin (cis-diamminedichloroplatinum II) remains one of the most powerful and widely used chemotherapeutic agents for treating various solid tumours (1, 2). Despite its clinical effectiveness, the therapeutic range of cisplatin is sharply limited by dose-dependent nephrotoxicity. Acute kidney injury (AKI) occurs in about 20 to 30% of patients receiving cisplatin therapy, often requiring dose reduction or early cessation of treatment, which can ultimately harm cancer outcomes (3, 4). While hydration therapy continues to be the standard preventive measure, it provides only partial protection, and specific pharmacological options approved to prevent or treat cisplatin-induced AKI (CI-AKI) are currently unavailable (5, 6). Therefore, the discovery of new renoprotective strategies is an urgent clinical need.

The pathophysiology of CI-AKI is intricate, involving an interplay amongst inflammation, vascular injury, and tubular epithelial cell death. While early research mainly focused on apoptosis and necrosis, growing evidence indicates that ferroptosis, an iron-dependent form of non-apoptotic regulated cell death, plays a crucial role in the development of cisplatin nephrotoxicity (7, 8). Ferroptosis is biochemically defined by the iron-driven build-up of harmful lipid peroxides and the depletion of antioxidant defences (9). Cisplatin accumulates mainly in proximal renal tubular cells, leading to mitochondrial dysfunction and the production of reactive oxygen species (ROS). This oxidative stress disturbs the System Xc-/GSH/GPX4 axis, a vital molecular regulator controlling lipid peroxidation (10, 11).

The specific biomarkers associated with this pathway are central to understanding renal injury. The suppression of GPX4 and the depletion of intracellular GSH lead to unchecked oxidation of polyunsaturated fatty acids, resulting in elevated toxic lipid peroxidation byproducts, such as malondialdehyde (MDA) and 4-hydroxynonenal (4-HNE), alongside an accumulation of labile ferrous iron (Fe^2+^) (12, 13). Functionally, these disturbances manifest as a rapid decline in the glomerular filtration rate, clinically identified through elevated serum creatinine (SCr) and blood urea nitrogen (BUN) levels (14).

In recent years, substantial preclinical research has examined the effectiveness of various synthetic agents and natural medicines, including traditional Chinese medicines (TCM), flavonoids, and polyphenols, in reducing CI-AKI (15–17). These studies suggest that such agents primarily protect the kidneys by inhibiting ferroptosis and restoring redox balance. However, despite encouraging individual reports, the data remains scattered and qualitatively varied. There is a critical lack of quantitative analysis of how these diverse interventions affect specific ferroptosis-related biomarkers across different experimental settings.

To address this gap, we conducted a multilevel meta-analysis of 58 preclinical studies (see the full list and their characteristics in Supplementary Files 1, 2, respectively). This study aims to provide a comprehensive mechanistic evaluation of the protective effects of synthetic and natural compounds on both traditional renal function markers and the critical biomarkers of the ferroptotic cascade. By synthesising this extensive dataset, we seek to clarify the mechanistic consensus underlying renoprotection and identify potential therapeutic candidates for future clinical translation.

## 2 Materials and methods

### 2.1 Search strategy and information sources

A systematic and comprehensive literature search was conducted across major electronic databases, including PubMed/MEDLINE and Web of Science, to identify relevant studies published between January 2020 and December 2025. The search strategy utilised a combination of Medical Subject Headings (MeSH) and free-text keywords with Boolean logic. The specific search string was: ((*cisplatin OR “cis-diamminedichloroplatinum” OR CDDP) AND (“kidney injury” OR nephrotoxicity OR “renal injury” OR AKI) AND (ferroptosis) AND (mice OR rat OR murine OR animal*)). Furthermore, the bibliographies of pertinent reviews and eligible articles were manually screened to identify any additional studies not captured by the electronic search. While this meta-analysis was not prospectively registered in an online database (e.g., PROSPERO), the study design, literature search, and data extraction protocols strictly adhered to the Preferred Reporting Items for Systematic Reviews and Meta-Analyses (PRISMA) 2020 guidelines (18).

### 2.2 Study selection process

**Figure 1:**
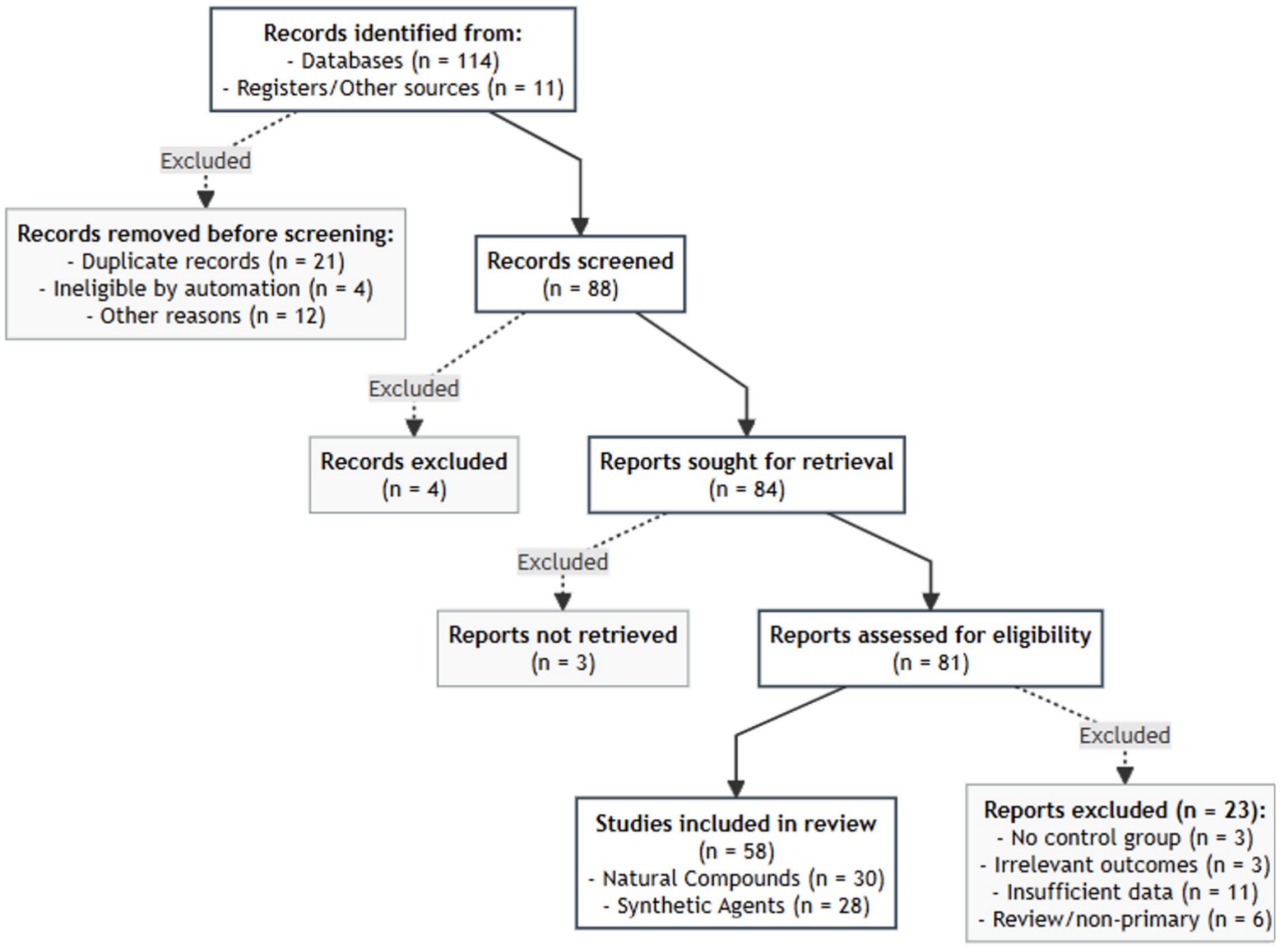
PRISMA 2020 flow chart.

The initial systematic search yielded a total of 125 records, comprising 114 from the primary electronic databases and 11 from registers and other sources. The complete step-by-step process of study selection and exclusion is illustrated in the PRISMA flow diagram (Figure 1). The 58 studies included in this meta-analysis represent a diverse range of experimental protocols.

Studies were selected for inclusion based on the PICOS (Population, Intervention, Comparator, Outcome, Study Design) framework:

- Population: *In vivo* rodent models (mice or rats) of CI-AKI induced by cisplatin.
- Intervention: Administration of natural medicines (e.g., flavonoids, polyphenols) or synthetic agents (e.g., Ferrostatin-1).
- Comparator: Control groups receiving cisplatin only.
- Outcome Measures: Quantitative data (mean and SD/SEM) for at least one renal marker (BUN/SCr) and one ferroptosis marker (GPX4, GSH, MDA, 4-HNE, or Fe^2+^).

Studies were explicitly excluded if they met any of the following criteria:

(1) Were clinical trials, reviews, case reports, or *in vitro*-only studies without *in vivo* validation.
(2) Used co-treatments that obstructed the identification of the specific drug’s effect.
(3) Did not offer extractable quantitative data (e.g., data shown only as images without scale bars or specific values).
(4) Used animals with pre-existing conditions (e.g., diabetic nephropathy) unrelated to cisplatin toxicity.

### 2.3 Data extraction and quality assessment

Two independent reviewers extracted data using a standardised form. Information collected included: bibliographic details (author, year), animal characteristics (species, sex, strain), cisplatin dosage and duration, intervention regimen (dose, route, duration), and quantitative outcome measures. The methodological quality of the included animal studies was evaluated using the risk-of-bias tool for animal studies (19). For studies presenting data solely in graphical formats, WebPlotDigitizer (Version 4.6, https://automeris.io/WebPlotDigitizer) was utilised to digitise and extract the numerical values. Where studies reported standard error (SE), these values were converted to SD prior to the meta-analysis.

### 2.4 Statistical analysis

#### 2.4.1 Analytical strategy

To reflect the inherent complexity of preclinical data, in which a single study often reports multiple outcomes (e.g., measuring both BUN and GPX4 in the same group of animals), a multilevel meta-analytical approach was used. This method avoids the issues of data aggregation or treating dependent effect sizes as independent, which would otherwise result in underestimated standard errors and inflated Type I error rates.

A random-effects framework was selected because substantial heterogeneity across studies was expected *a priori*. The included studies differed in multiple key aspects, including animal species (mice vs. rats), cisplatin dosage and administration protocols, intervention types (natural compounds vs. synthetic agents), and outcome measures spanning both functional biomarkers (e.g., BUN, creatinine) and mechanistic indicators of ferroptosis (e.g., GPX4, GSH, MDA). These variations imply that the true underlying effect sizes are unlikely to be identical across studies but instead follow a distribution. Therefore, a random-effects model was considered more appropriate than a fixed-effects model, as it accounts for both within-study sampling error and between-study variability, enabling inference beyond the included studies to a broader population of experimental conditions.

#### 2.4.2 Effect size definition

The primary outcome measure was the Standardised Mean Difference (SMD), specifically calculated as Hedges’ g (20). This metric was chosen to standardise data across different units of measurement and to correct for the inherent upward bias in effect size estimates often found with small sample sizes (N < 10), typical of preclinical research. The effect size was calculated using the following formula:


$$g=(1-\frac{3}{4(n_{1}+n_{2})-9})*(\frac{x_{\text{treat}}-x_{\text{model}}}{s^{*}})$$

where $x_{\text{treat}}$ and $x_{\text{model}}$ are the mean values of the treatment and cisplatin-model groups, respectively, n_1_ and n_2_ represent the sample sizes, and $s^{*}$ denotes the pooled standard deviation.

#### 2.4.3 Sampling variance assumption

Because correlations amongst multiple outcomes within the same study were rarely reported, dependency amongst effect sizes was addressed using a multilevel random-effects structure with random intercepts at both the study and effect-size levels. This approach accounts for within-study dependence by explicitly modelling the hierarchical structure of the data, without requiring direct specification of correlations amongst sampling errors.

#### 2.4.4 Multilevel model specification

A three-level random-effects model was designed to partition the total heterogeneity into sampling variance, within-study variance, and between-study variance (21, 22):

Level 1: Sampling variance (at the individual effect size level).
Level 2: Within-study variance (variance amongst different outcomes within the same study).
Level 3: Between-study variance (variance across different research papers).

#### 2.4.5 Meta-regression analyses

To explore the sources of heterogeneity and the moderation of treatment effects, multilevel meta-regressions were carried out. Two separate models were developed for Natural and Synthetic agents. The primary moderator was Outcome Type, with BUN serving as the reference category to which other biomarkers (Creatinine, GSH, GPX4, Fe^2+^, MDA, and 4-HNE) were compared.

#### 2.4.6 Robust inference

To ensure robust statistical inference in the presence of dependent effect sizes, we employed cluster-robust variance estimation (CRVE) with clustering at the study level. This approach yields standard errors and confidence intervals that are robust to potential misspecification of the within-study dependence structure. Statistical significance was evaluated using small-sample adjustments based on the Satterthwaite approximation (23).

#### 2.4.7 Interpretation and scope

The interpretation of the resulting *β* coefficients centres on the magnitude and direction of change relative to the cisplatin-only model group. A negative *β* for markers such as BUN or MDA indicates a therapeutic reduction in injury or oxidative stress, while a positive *β* for markers such as GPX4 or GSH indicates a successful recovery of antioxidant defences. The scope of this analysis is confined to *in vivo* rodent models of CI-AKI and should be regarded as a foundational synthesis for future clinical translation. Importantly, the direction of the *β* coefficients must be understood in the context of each biomarker’s biological function. While negative *β* values for functional markers (BUN, Creatinine) and lipid peroxidation products (MDA, 4-HNE) reflect a therapeutic reduction in renal injury, the markedly positive shifts observed for GSH and GPX4 indicate a strong restoration of the antioxidant defence system. Since cisplatin treatment usually causes significant depletion of these antioxidants, a larger positive effect size in these categories demonstrates a successful reversal of cisplatin-induced biochemical suppression, restoring the cellular state towards physiological homeostasis.

Heterogeneity was evaluated using the *Q* statistic and *I^2^* values. To ensure robust results, Cluster-Robust Variance Estimation (CRVE) with the Satterthwaite approximation was applied to all meta-regression analyses. Publication bias was assessed via the multilevel version of Egger’s test and the Trim and Fill method. All analyses were performed in R using the metafor and clubSandwich packages.

## 3 Results

### 3.1 Study selection process

A comprehensive systematic search across PubMed/MEDLINE and Web of Science databases, supplemented by registers and other sources, initially yielded 125 records (114 from databases and 11 from other sources). After removing records prior to screening (21 duplicates, 4 ineligibles identified by automation, and 12 for other reasons), 88 records were screened by title and abstract. Of these, 84 reports were sought for retrieval, and 81 were assessed for full-text eligibility.

After applying the PICOS framework, 58 unique preclinical studies met all eligibility criteria (see Supplementary File 2 for a summary of these studies) and were included in the quantitative meta-analysis. These studies were categorised into two primary intervention groups as follows:

- Natural Compounds: 30 studies providing 145 effect sizes (k), investigating the efficacy of flavonoids, polyphenols, and herbal extracts.
- Synthetic Agents: 28 studies providing 120 effect sizes (k), focusing on specific ferroptosis inhibitors (e.g., Ferrostatin-1), iron chelators, and mitochondria-targeted antioxidants.

The selection process, including reasons for exclusion at the full-text stage (such as lack of *in vivo* data, missing standard deviations, or absence of specific ferroptosis biomarkers), is summarised in the PRISMA flow diagram (Figure 1).

### 3.2 Characteristics of included studies

The 58 studies included in this meta-analysis represent a diverse range of experimental protocols. Most studies utilised mice (72%), primarily the C57BL/6 strain, while the remainder utilised rats (28%), mostly Sprague–Dawley or Wistar strains. Cisplatin doses ranged from 10 to 25 mg/kg, typically administered as a single intraperitoneal injection to induce AKI within a 48- to 72-h window. The therapeutic interventions were categorised into Natural Compounds (e.g., Baicalein, Quercetin, Morroniside) and Synthetic Agents (e.g., Ferrostatin-1, JP4-039, SKQ1). Outcomes were balanced across renal function markers (BUN/SCr) and the “ferroptotic triad”: iron accumulation, lipid peroxidation (MDA, 4-HNE), and antioxidant depletion (GPX4, GSH).

### 3.3 Meta-regression results for natural agents

As shown in Table 1, natural compounds exerted a significant renoprotective effect, particularly in reducing BUN levels (Intercept *β* = −3.84, *p* < 0.001). The multilevel meta-regression revealed that “Outcome Type” was a powerful moderator (Q_M_ = 605.34, *p* < 0.001). Specifically, the restoration of antioxidant defences GSH (*β* = 6.99) and GPX4 (*β* = 8.56) showed significantly larger effect sizes than the reference category (BUN). In contrast, the effects on Creatinine, Fe^2+^, MDA, and 4-HNE did not deviate significantly from the baseline improvement observed in BUN (*p* > 0.05).

**Table 1:** Outcome-type moderation of treatment effects for natural agents.

Predictor	Estimate (*β*)	SE	*z*	*p*	95% CI
BUN	−3.84***	0.28	−13.88	<0.001	[−4.38, −3.30]
Creatinine	−0.31	0.39	−0.78	0.433	[−1.08, 0.46]
GSH	6.99***	0.46	15.52	<0.001	[6.26, 8.07]
GPX4	8.56***	0.55	17.54	<0.001	[8.63, 10.80]
Fe^2+^	0.62	0.56	1.10	0.270	[−0.48, 1.73]
MDA	0.54	0.43	1.27	0.204	[−0.29, 1.38]
4-HNE	0.28	0.83	0.34	0.736	[−1.35, 1.90]

**Outcome:** Hedges’ g (Treat − Model).

**Sample:** Natural treatment compounds only.

**Model:** Multilevel random-effects meta-regression (random intercepts for study and effect size).

**Reference category:** BUN.

Estimates (*β*) represent the difference in effect size relative to the reference category (BUN). A negative intercept indicates a reduction in injury markers, while positive estimates for GSH and GPX4 indicate significant restoration of antioxidant defences. *** *p* < 0.001.

### 3.4 Meta-regression results for synthetic agents

The findings for synthetic agents mirrored those of natural compounds (Table 2). Synthetic interventions significantly blunted the rise in BUN (Intercept *β* = −4.06, *p* < 0.001). Outcome type again served as a robust moderator (Q_M_ = 509.17, *p* < 0.001), with GSH (*β* = 6.33) and GPX4 (*β* = 8.71) showing the largest positive effects, indicating a primary mechanism centred on restoring the antioxidant shield.

**Table 2:** Outcome-type moderation of treatment effects for synthetic agents.

Predictor	Estimate (*β*)	SE	*z*	*p*	95% CI
BUN	−4.06***	0.32	−12.59	<0.001	[−4.61, −3.37]
Creatinine	−0.12	0.40	−0.26	0.797	[−0.89, 0.68]
GSH	6.33***	0.52	12.88	<0.001	[5.70, 7.75]
GPX4	8.71***	0.54	17.66	<0.001	[8.48, 10.60]
Fe^2+^	0.58	0.82	0.70	0.482	[−1.03, 2.19]
MDA	0.40	0.47	0.84	0.399	[−0.52, 1.31]
4-HNE	0.41	0.74	0.56	0.575	[−1.03, 1.86]

**Outcome:** Hedges’ g (Treat − Model).

**Sample:** Synthetic treatment compounds only.

**Model:** Multilevel random-effects meta-regression (random intercepts for study and effect size).

**Reference category:** BUN.

Estimates (*β*) represent the difference in effect size relative to the reference category (BUN). A negative intercept indicates a reduction in injury markers, while positive estimates for GSH and GPX4 indicate significant restoration of antioxidant defences. *** *p* < 0.001.

### 3.5 Heterogeneity and publication Bias

The overall pooled analysis (k = 265; 58 studies) revealed statistically significant total heterogeneity (*Q* = 3174.00, *p* < 0.001). Similar patterns were observed in subgroup analyses: for natural compounds (k = 145; 30 studies), *Q* = 1832.00, *p* < 0.001; and for synthetic compounds (k = 120; 28 studies), *Q* = 1338.00, *p* < 0.001. It should be noted that in three-level meta-analytic models, the conventional *I^2^* statistic can be misleadingly low because total variance is partitioned across sampling, within-study, and between-study levels. The significant Q statistics confirm substantial heterogeneity across the dataset, largely attributable to the outcome-type moderator (*Q_M_* > 500, *p* < 0.001 in both models). Multilevel Egger’s tests indicated slight publication bias for natural agents (*p* = 0.042), whereas synthetic agents were less affected (*p* = 0.078). However, “Trim and Fill” analysis confirmed that the core conclusions remain statistically robust even after adjusting for missing studies. The methodological quality and risk of bias of the included primary studies were assessed using the Cochrane Risk of Bias tool, adapted for these studies (see Supplementary File 3).

**Model statistics:**

- *k* = 145 effect sizes, 30 studies.
- Test of moderators: *Q_M_* (6) = 605.34, *p* < 0.001.
- Residual heterogeneity: *Q_E_* (138) = 607.88, *p* < 0.001.

To explore whether the treatment effects of natural compounds differ across biomarker categories, we performed a multilevel meta-regression with outcome type as a moderator. The model accounted for dependencies amongst effect sizes by including random intercepts at both the study and effect-size levels.

The intercept indicated a large and statistically significant negative effect for the reference outcome category (*β* = −3.84, *p* < 0.001), suggesting that natural treatments substantially reduced biomarker levels relative to the cisplatin model condition for this outcome type.

Outcome type emerged as a strong moderator of treatment effects (*Q_M_* (6) = 605.34, *p* < 0.001). Specifically, GSH and GPX4 were associated with markedly larger positive shifts in effect sizes compared with the reference category (*β* = 6.99 and *β* = 8.56, respectively; both *p* < 0.001). In contrast, Creatinine, Fe^2+^, MDA and 4-HNE did not differ significantly from the reference category. All forest plots are provided in Supplementary File 4.

These results indicate that, amongst natural compounds, treatment effectiveness varies substantially across biomarkers, with particularly pronounced effects observed for GSH and GPX4.

**Figure 2:**
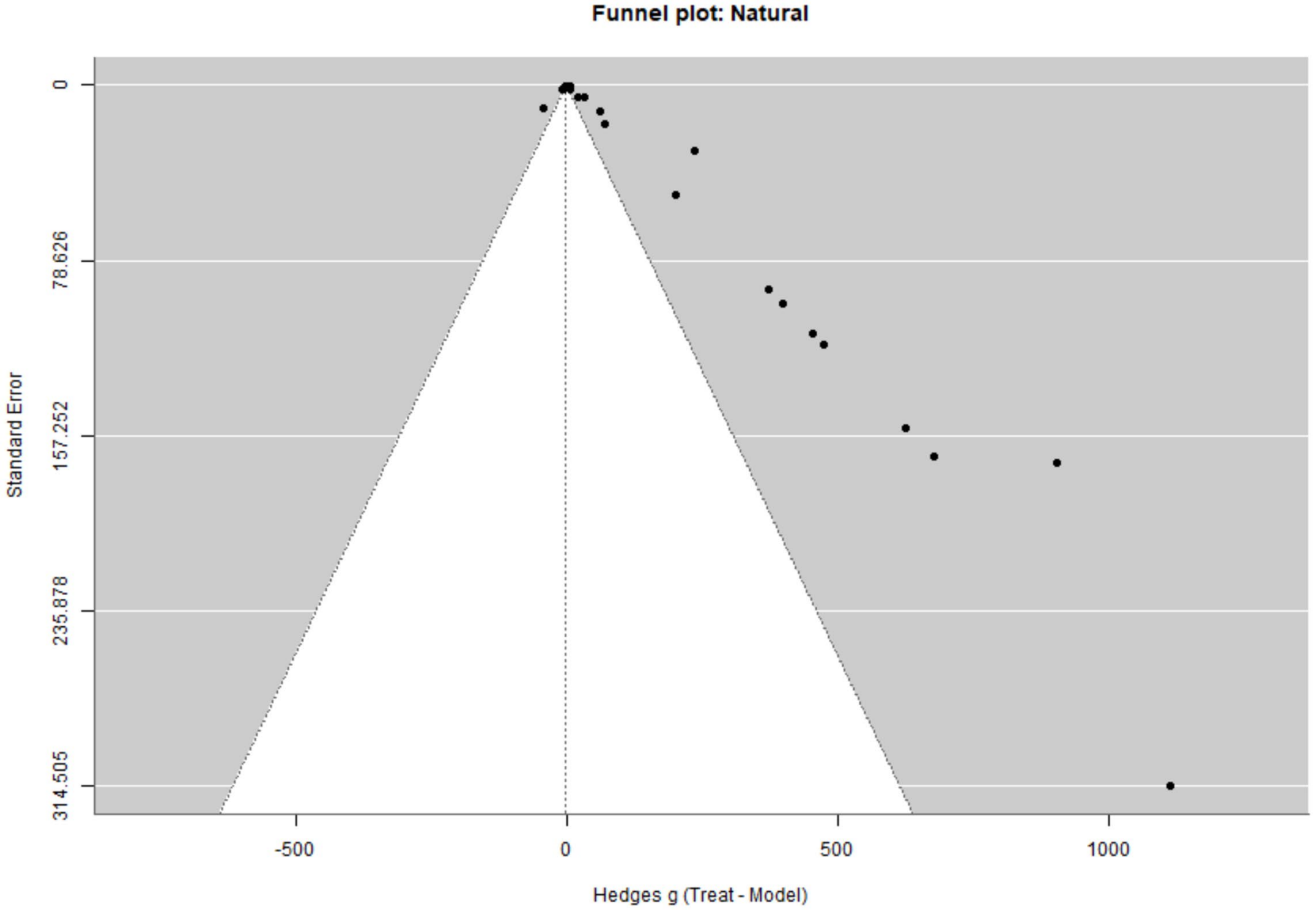
Funnel plot of treatment effects for natural compounds.

Visual inspection of the funnel plot in Figure 2 indicates asymmetry, consistent with potential small-study effects. Given the strong outcome-type heterogeneity and dependent effect sizes, this pattern is interpreted as exploratory rather than definitive evidence of publication bias.

**Model statistics:**

- *k* = 120 effect sizes, 28 studies.
- Test of moderators: *Q_M_* (6) = 509.17, *p* < 0.001.
- Residual heterogeneity: *Q_E_* (113) = 467.97, *p* < 0.001.

A parallel multilevel meta-regression was conducted for studies employing synthetic compounds to examine whether treatment effects varied across biomarker categories. As in the natural-compound analysis, random intercepts were specified at the study and effect-size levels.

The intercept indicated a large, statistically significant negative effect for the reference outcome category (*β* = −4.06, *p* < 0.001), indicating that synthetic treatments substantially reduced biomarker levels relative to the cisplatin model condition.

The overall pooled analysis (k = 265; 58 studies) revealed statistically significant heterogeneity (*Q* = 3174.00, *p* < 0.001), with similar patterns for natural compounds (*Q* = 1832.00, *p* < 0.001) and synthetic compounds (Q = 1338.00, *p* < 0.001). As discussed in Section 3.5, the low conventional *I^2^* values observed in three-level models should not be interpreted as evidence of low heterogeneity, given the partitioning of variance across multiple levels.

Outcome type again emerged as a strong moderator of treatment effects (*Q_M_* (6) = 509.17, *p* < 0.001). GSH and GPX4 showed markedly larger effect sizes than the reference category (*β* = 6.33 and *β* = 8.71, respectively; both *p* < 0.001), whereas Creatinine, Fe^2+^, MDA and 4-HNE did not differ significantly.

Overall, the pattern of moderation closely mirrored that observed for natural compounds.

**Figure 3:**
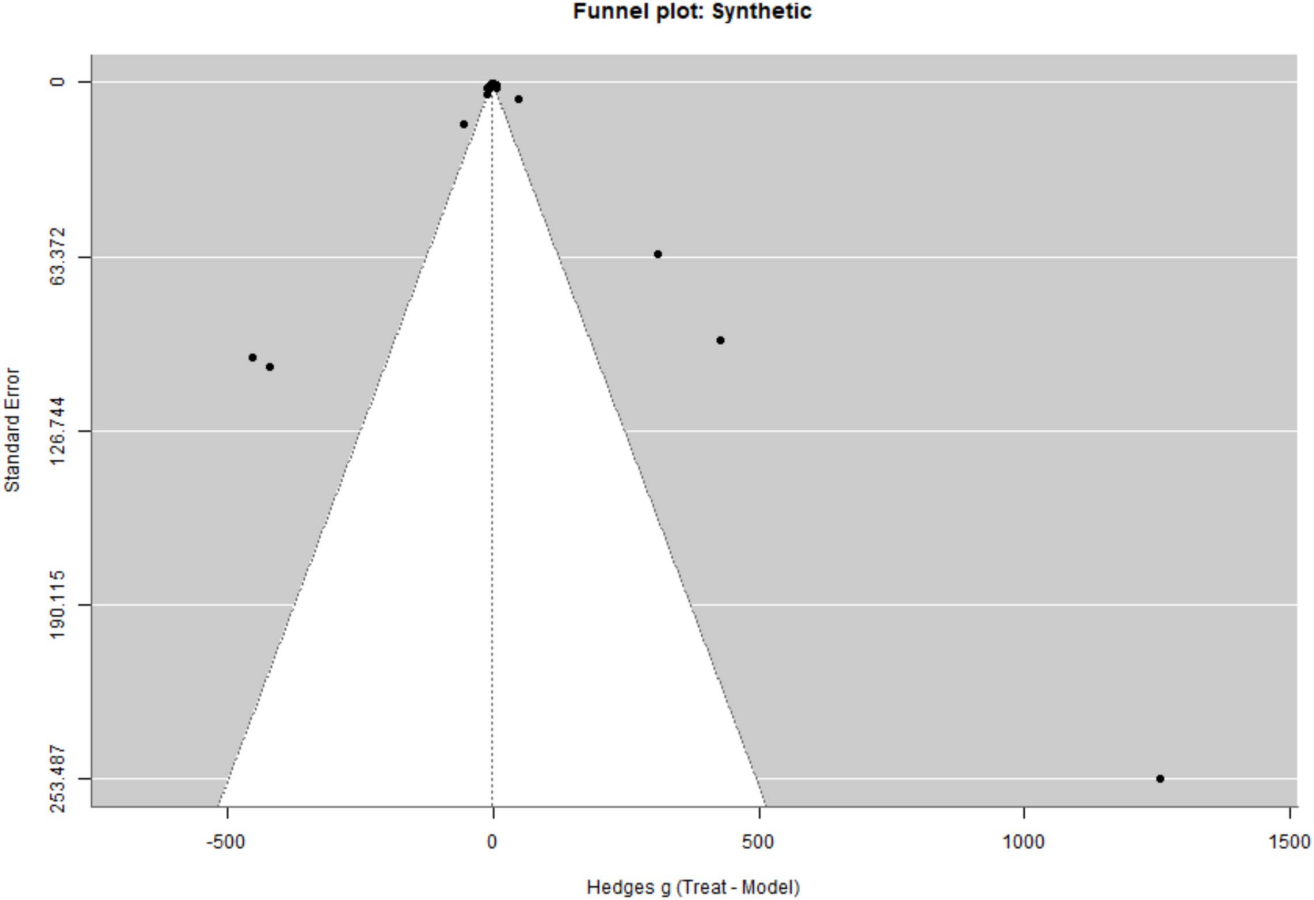
Funnel plot of treatment effects for synthetic compounds.

The funnel plot in Figure 3 for synthetic compounds also displays visible asymmetry, with several imprecise studies reporting relatively large positive effect sizes. This pattern is consistent with potential small-study effects. However, as with the natural compounds, the observed asymmetry should be interpreted cautiously, given the substantial heterogeneity across biomarker types and the presence of multiple dependent effect sizes within studies.

To further assess potential publication bias, we applied PET–PEESE models. The PET model indicated a positive and statistically significant intercept (*β* = 1.29, *p* = 0.031), along with a significant negative association between effect sizes and their standard errors, suggesting small-study effects. The PEESE model, which provides a bias-corrected estimate, yielded a negative effect (*β* = −1.61, *p* < 0.001). Taken together, these findings indicate that the magnitude of the observed effects may be inflated and should be interpreted with caution.

For the overall dataset, Egger’s regression test indicated significant funnel-plot asymmetry (z = −5.59, *p* < 0.001), suggesting the presence of small-study effects. The corresponding limit estimate as the standard error approached zero was *β* = 1.29, 95% CI [0.12, 2.45].

When examined by subgroup, funnel-plot asymmetry was also evident for natural treatments (z = −2.90, *p* = 0.004) and for synthetic treatments (z = −4.82, *p* < 0.001). The limit estimates were *β* = 0.89 (95% CI: [−0.91, 2.69]) for natural treatments and *β* = 1.50 (95% CI: [−0.07, 3.07]) for synthetic treatments. Taken together, these findings suggest potential small-study effects, so the magnitude of the pooled effects should be interpreted with caution.

To address the comparative efficacy between these two therapeutic classes, we first evaluated the pooled effect sizes and 95% confidence intervals (CIs) derived from our independent multilevel meta-regression models. For natural compounds, the overall standardised effect size for the reduction of the primary injury marker (BUN) was *β* = −3.84 (95% CI: −4.38 to −3.30, *p* < 0.001). Synthetic compounds demonstrated a nearly identical pooled effect size for BUN reduction (*β* = −4.06, 95% CI: −4.61 to −3.37, *p* < 0.001). The moderation effects for the restoration of critical antioxidant defences were also highly comparable; the effect size shifts for GPX4 were *β* = 8.56 (95% CI: 8.63 to 10.80) for natural agents and *β* = 8.71 (95% CI: 8.48 to 10.60) for synthetic agents, while GSH shifts were *β* = 6.99 (95% CI: 6.26 to 8.07) and *β* = 6.33 (95% CI: 5.70 to 7.75), respectively. The 95% CIs of these two classes substantially overlap across all major functional and mechanistic biomarkers.

To formally test this comparison, we included treatment type (natural vs. synthetic) as a moderator in the multilevel meta-regression. The results confirmed that the difference between natural and synthetic compounds was not statistically significant (*β* = −0.29, *p* = 0.578). Therefore, both natural and synthetic interventions provide statistically comparable mitigation of acute kidney injury in preclinical CI-AKI models. Consequently, the choice between natural and synthetic ferroptosis inhibitors in future clinical applications may depend more on factors such as drug bioavailability, toxicity profiles, and timing of administration than on absolute differences in renoprotective efficacy.

## 4 Discussion

The clinical usefulness of cisplatin has historically been limited by its dose-dependent nephrotoxicity, affecting up to 30% of patients (2). Although oxidative stress has long been regarded as a cause of injury, the failure of non-specific antioxidants in clinical trials indicates a need to target specific programmed cell death pathways rather than random damage. This meta-analysis, which combines 265 effect sizes from 58 high-quality preclinical studies, provides the first quantitative evidence that inhibiting ferroptosis is a highly effective means of protecting the kidneys. Our multilevel meta-regression shows that while both natural and synthetic agents significantly reduce functional impairment (Intercepts *β* = −3.84 and −4.06, respectively), the most notable therapeutic improvements are seen in restoring the antioxidant defence system, shifting the focus from passive radical scavenging to active metabolic rewiring.

### 4.1 The hierarchical mechanism: GPX4 as the master regulator

The most notable finding of this study is the hierarchical sensitivity of biomarkers to ferroptosis inhibition. The meta-regression identified “Outcome Type” as a significant moderator (*Q_M_* > 500), with the restoration of GPX4 (*β* = 8.56 for natural; 8.71 for synthetic) and GSH (*β* = 6.99 for natural; 6.33 for synthetic) exerting greater effects than those of other markers.

This magnitude of effect size—approaching nearly 10 standard deviations—indicates that ferroptosis inhibitors do not simply “blunt” damage; they fundamentally reconfigure the System Xc−/GSH/GPX4 axis. In the context of CI-AKI, cisplatin triggers a “ferroptotic storm” by depleting intracellular GSH and inactivating GPX4, the only enzyme capable of neutralising lethal phospholipid hydroperoxides (10). Our results confirm that the primary therapeutic signature of these compounds is the active replenishment of this axis, which prevents the unchecked oxidation of polyunsaturated fatty acids before membrane rupture occurs.

### 4.2 Functional restoration and the clinical benchmark

The clinical benchmark for any CI-AKI intervention is the stabilisation of glomerular filtration markers. Our analysis confirms that ferroptosis inhibitors significantly reduce the precipitous rise in Blood Urea Nitrogen (BUN) (*β* = −4.0) and Serum Creatinine. This functional restoration is not merely a transient biochemical shift but reflects the preservation of renal cortical architecture.

Cisplatin-induced AKI often triggers “maladaptive repair,” characterised by tubular cast formation and basement membrane thickening, which acts as a gateway to chronic kidney disease (CKD) (24). We hypothesise that by stabilising mitochondrial membranes and preventing early tubular necrosis, inhibitors such as ADAMTS-13 and SKQ1 might also mitigate subsequent upregulation of fibrotic markers such as Collagen 1a1 and Fibronectin (25). Based on this functional restoration, future longitudinal studies should investigate whether the therapeutic window for ferroptosis inhibition provides a prophylactic shield against the AKI-to-CKD transition.

### 4.3 Iron sequestration vs. lipid peroxidation

The analysis of the “ferroptotic triad”—iron accumulation, lipid peroxidation, and antioxidant depletion—revealed a nuanced therapeutic profile. While MDA and 4-HNE showed smaller relative shifts than GPX4, their reductions remained statistically significant and aligned with functional recovery. This suggests that lipid peroxidation products are downstream effectors of the death signal, whereas the GPX4/GSH axis functions as the upstream decision-maker.

Furthermore, iron dysregulation, often caused by ferritinophagy (NCOA4-mediated degradation of ferritin), was effectively reduced by agents such as bergenin and morroniside (6, 26). The success of iron-focused strategies emphasises the role of the Fenton reaction as the “metallic catalyst” of CI-AKI. This is supported by foundational rodent models demonstrating that labile iron accumulation is a prerequisite for cisplatin-induced tubular necrosis, and that iron-specific chelation can effectively halt the progression of renal damage (27). Our discovery that mitochondrial-targeted agents such as SKQ1 act with high precision highlights the importance of protecting the mitochondrial labile iron pool to prevent ferroptotic triggers (28).

### 4.4 The parity of natural and synthetic interventions

A high-impact finding of this study is the statistical parity between natural medicines (e.g., flavonoids, TCM extracts) and high-precision synthetic agents (*p* > 0.05). This suggests that the “metabolic rewiring” provided by natural polyphenols is functionally equivalent to the “organelle-targeted shielding” of synthetic inhibitors (29).

- Synthetic Agents: Molecules such as JP4-039 offer surgical precision, targeting mitochondrial ROS or specific enzymes, such as ACSL4, to provide a direct upstream blockade of the ferroptotic cascade (24).
- Natural Agents: Compounds such as morroniside and salvianolic acid B utilise multi-modal pathways. Specifically, salvianolic acid B has been shown to target PRDX5 to neutralise mitochondrial peroxides, providing an organelle-specific defence comparable to synthetic inhibitors (30). Furthermore, these agents often involve repair of the gut-kidney axis, p53-mediated regulation of SLC7A11 (5, 26), and glutathione metabolism rewiring that confers dual protection against both apoptosis and ferroptosis (31).

This parity opens a significant clinical door for the repurposing of natural compounds as cost-effective, low-toxicity adjuvant therapies for cancer patients.

### 4.5 Signalling crosstalk: from ferroptosis to PANoptosis

Our study shows that ferroptosis is not an isolated event but a trigger for systemic renal inflammation. The release of damage-associated molecular patterns (DAMPs) from ferroptotic tubules activates the IL-6/JAK/STAT3 axis, creating a feedback loop that amplifies injury (19).

Emerging evidence suggests that CI-AKI involves PANoptosis—a complex interplay of apoptosis, necroptosis, and ferroptosis. Interventions like amentoflavone offer a comprehensive profile by modulating Nrf2 and simultaneously inhibiting these interconnected death pathways (32, 33). While the GPX4 axis is central, recent findings indicate that metabolic disturbances, such as the IDH1-R132H mutation, can aggravate injury by disrupting the interaction between NDUFA1 and FSP1, thereby promoting ferroptotic vulnerability (34). This integrated mechanism likely accounts for the strong efficacy observed across our dataset, as these agents arrest the primary ferroptotic insult while reducing the secondary inflammatory response.

### 4.6 Limitations and the translation gap

Despite the robust statistical evidence, the high residual heterogeneity (*Q_E_* > 400) indicates that the magnitude of renoprotection is influenced by animal species, cisplatin dosage (10–25 mg/kg), and the timing of intervention. While the therapeutic window identified in mouse models (effective up to 24 h post-insult) is promising, clinical diagnosis in humans is often delayed, presenting a challenge for translation. Furthermore, significant residual heterogeneity was observed across our models. While outcome type accounted for a large portion of the variance, other experimental parameters, such as variations in cisplatin dosage (ranging from 10 to 25 mg/kg), animal species (mice vs. rats), and the timing of drug administration (pre-treatment vs. post-injury), undoubtedly contribute to this heterogeneity. Because the literature is heavily skewed toward specific models (e.g., 20 mg/kg doses in C57BL/6 mice), conducting a statistically powered meta-regression on these sub-variables was not feasible. Future primary studies should standardise these models to better isolate the influence of dosage and timing on ferroptotic renoprotection.

Furthermore, the strict focus of this meta-analysis on murine models limits the generalizability of the findings across the broader animal kingdom. While mice and rats provide highly standardised mammalian frameworks for CI-AKI, thereby limiting unmanageable baseline physiological variance in our quantitative synthesis, future systematic reviews should expand this scope. Exploring alternative experimental models, such as zebrafish or guinea pigs, may offer unique and valuable insights into the evolutionary conservation of ferroptotic pathways and their role in nephrotoxicity.

Finally, our Risk of Bias assessment (Supplementary Table S3) revealed that while attrition and reporting biases were low, critical domains such as selection bias (randomisation details) and detection bias (blinding) were predominantly “Unclear” across the included studies. Consistent with broader challenges in preclinical research, this pervasive lack of methodological reporting limits the absolute certainty of the pooled estimates and may contribute to an overestimation of treatment effects, underscoring the need for stricter adherence to guidelines in future CI-AKI models.

## 5 Conclusion

This systematic review and multilevel meta-analysis of 58 preclinical studies confirm that targeting ferroptosis is a highly effective and dependable strategy for reducing cisplatin-induced acute kidney injury. The mechanistic consensus from our quantitative synthesis is that the therapeutic prevention of CI-AKI mainly relies on the active restoration of the System Xc−/GSH/GPX4 axis rather than passive antioxidant scavenging.

Our findings show that both natural polyphenols and synthetic mitochondrial antioxidants provide comparable kidney protection, effectively reducing increases in Blood Urea Nitrogen and Serum Creatinine while preventing iron-catalysed lipid peroxidation, which causes tubular necrosis. The large positive effect sizes observed for GPX4 and GSH (*β* up to 8.71) highlight these biomarkers as the most sensitive and vital indicators of therapeutic success in the context of CI-AKI. From a clinical perspective, these results suggest that ferroptosis inhibition may serve as a promising strategy for preventing acute tubular damage. Whether this acute protection can also attenuate the long-term progression from AKI to chronic kidney disease remains an important hypothesis warranting investigation in future longitudinal preclinical and clinical studies. To facilitate clinical translation, future research should prioritise:

1. Dual-Targeting Strategies: Combining iron sequestration with GPX4 stabilisation to provide a “double-lock” against ferroptotic triggers.
2. Clinical Timing: Validating the efficacy of these agents in late-stage intervention models that more accurately reflect human AKI diagnosis.
3. Oncological Safety: Confirming that ferroptosis-targeted renoprotection does not interfere with the primary tumour-killing efficacy of cisplatin.

Ultimately, incorporating ferroptosis inhibitors into cisplatin-based chemotherapy regimens may expand the therapeutic window of this vital drug, ensuring that life-saving cancer treatment is no longer hindered by irreversible renal toxicity (35, 36).

## Data Availability

The original contributions presented in the study are included in the article/Supplementary material, further inquiries can be directed to the corresponding author.

## References

[1] Dasari S Tchounwou PB. Cisplatin in cancer therapy: molecular mechanisms of action. Eur J Pharmacol. (2014) 740:364–78. doi: 10.1016/j.ejphar.2014.07.025 25058905 PMC4146684

[2] Kim DH Choi HI Park JS Kim CS Bae EH Ma SK . Farnesoid X receptor protects against cisplatin-induced acute kidney injury by regulating the transcription of ferroptosis-related genes. Redox Biol. (2022) 54:102382. doi: 10.1016/j.redox.2022.102382, 35767918 PMC9241134

[3] Guo S Zhou L Liu X Gao L Li Y Wu Y. Baicalein alleviates cisplatin-induced acute kidney injury by inhibiting ALOX12-dependent ferroptosis. Phytomedicine. (2024) 130:155757. doi: 10.1016/j.phymed.2024.155757, 38805781

[4] Li Y Li K Zhao W Wang H Xue X Chen X . VPA improves ferroptosis in tubular epithelial cells after cisplatin-induced acute kidney injury. Front Pharmacol. (2023) 14:1147772. doi: 10.3389/fphar.2023.1147772, 37153759 PMC10155836

[5] Jin X He R Lin Y Liu J Wang Y Li Z . Shenshuaifu granule attenuates acute kidney injury by inhibiting ferroptosis mediated by p53/SLC7A11/GPX4 pathway. Drug Des Devel Ther. (2023) 17:3363–83. doi: 10.2147/DDDT.S433994, 38024532 PMC10656853

[6] Hu J Zhang Y Zhang Y Shi N Miu Y Huang J . Bergenin inhibits ferritinophagy and ferroptosis in cisplatin-induced acute kidney injury by activating the p-GSK3β/Nrf2/PPARγ pathway. Int Immunopharmacol. (2025) 147:114004. doi: 10.1016/j.intimp.2024.114004 39793228

[7] Hu Z Zhang H Yi B Yang S Liu J Hu J . VDR activation attenuate cisplatin induced AKI by inhibiting ferroptosis. Cell Death Dis. (2020) 11:73. doi: 10.1038/s41419-020-2256-z, 31996668 PMC6989512

[8] Mishima E Sato E Ito J Yamada KI Suzuki C Oikawa Y . Drugs repurposed as antiferroptosis agents suppress organ damage, including AKI, by functioning as lipid peroxyl radical scavengers. J Am Soc Nephrol. (2020) 31:280–96. doi: 10.1681/ASN.2019060570, 31767624 PMC7003311

[9] Dixon SJ Lemberg KM Lamprecht MR Skouta R Zaitsev EM Gleason CE . Ferroptosis: an iron-dependent form of nonapoptotic cell death. Cell. (2012) 149:1060–72. doi: 10.1016/j.cell.2012.03.042, 22632970 PMC3367386

[10] Pan M Wang Z Wang Y Jiang X Fan Y Gong F . Celastrol alleviated acute kidney injury by inhibition of ferroptosis through Nrf2/GPX4 pathway. Biomed Pharmacother. (2023) 166:115333. doi: 10.1016/j.biopha.2023.115333 37598476

[11] Liang NN Guo YY Zhang XY Ren YH He YZ Liu ZB . Mitochondrial dysfunction-evoked DHODH acetylation is involved in renal cell ferroptosis during cisplatin-induced acute kidney injury. Adv Sci. (2024) 11:2404753. doi: 10.1002/advs.202404753, 39303219 PMC11578349

[12] Zhang Z Zhou H Gu W Wei Y Mou S Wang Y . CGI1746 targets σ1R to modulate ferroptosis through mitochondria-associated membranes. Nat Chem Biol. (2024) 20:699–709. doi: 10.1038/s41589-023-01512-1 38212578

[13] Zhu Z Liu X Li P Wang H Zhang Y Liu M . Renal clearable quantum dot–drug conjugates modulate labile iron species and scavenge free radicals for attenuating chemotherapeutic drug-induced acute kidney injury. ACS Appl Mater Interfaces. (2023) 15:21854–65. doi: 10.1021/acsami.3c00714, 37115671

[14] Ronco C Bellomo R Kellum JA. Acute kidney injury. Lancet. (2019) 380:756–66. doi: 10.1016/S0140-6736(11)61454-2 22617274

[15] Song Z Li Z Pan T Liu T Gong B Wang Z . Protopanaxadiol prevents cisplatin-induced acute kidney injury by regulating ferroptosis. J Pharm Pharmacol. (2024) 76:884–96. doi: 10.1093/jpp/rgae050, 38708970

[16] Cai F Li D Zhou K Zhang W Yang Y. Tiliroside attenuates acute kidney injury by inhibiting ferroptosis by disrupting the NRF2-KEAP1 interaction. Phytomedicine. (2024) 126:155407. doi: 10.1016/j.phymed.2024.155407 38340577

[17] Dai Q Xiang Y Qiang R Li G Song Y Yu Y . Aloe-emodin mitigates cisplatin-induced acute kidney injury by Nrf2-mediated ferroptosis regulation. Free Radic Biol Med. (2025) 241:104–16. doi: 10.1016/j.freeradbiomed.2025.09.016, 40947035

[18] Page MJ McKenzie JE Bossuyt PM Boutron I Hoffmann TC Mulrow CD . The PRISMA 2020 statement: an updated guideline for reporting systematic reviews. BMJ. (2021) 372:n71. doi: 10.1136/bmj.n71, 33782057 PMC8005924

[19] Hooijmans CR Rovers MM de Vries RB Leenaars M Ritskes-Hoitinga M Langendam MW. SYRCLE’S risk of bias tool for animal studies. BMC Med Res Methodol. (2014) 14:43. doi: 10.1186/1471-2288-14-43, 24667063 PMC4230647

[20] Hedges LV Olkin I. Statistical Methods for Meta-Analysis. Orlando: Academic Press (1985).

[21] Viechtbauer W. Conducting meta-analyses in R with the metafor package. J Stat Softw. (2010) 36:1–48. doi: 10.18637/jss.v036.i03

[22] Assink M Wibbelink CJ. Fitting three-level meta-analytic models in R: a step-by-step tutorial. Quant Meth Psychol. (2016) 12:154–74. doi: 10.20982/tqmp.12.3.p154

[23] Pustejovsky JE Tipton M. Small-sample methods for cluster-robust variance estimation and hypothesis testing in fixed effects models. J Bus Econ Stat. (2018) 36:672–83. doi: 10.1080/07350015.2016.1247004

[24] Airik M Clayton K Wipf P Airik R. JP4-039 mitigates cisplatin-induced acute kidney injury by inhibiting oxidative stress and blocking apoptosis and Ferroptosis in mice. Antioxidants. (2024) 13:1534. doi: 10.3390/antiox13121534, 39765862 PMC11727076

[25] Meng X Huang W Mo W Shu T Yang H Ning H. ADAMTS-13-regulated nuclear factor E2-related factor 2 signaling inhibits ferroptosis to ameliorate cisplatin-induced acute kidney injury. Bioengineered. (2021) 12:11610–21. doi: 10.1080/21655979.2021.1994707 34666603 PMC8810018

[26] Li H Xu K Mao W Yu B Liu Z Huang F . Morroniside alleviates cisplatin-induced renal injury and gut dysbiosis via the gut–kidney axis and ferroptosis. Int Immunopharmacol. (2025) 153:114430. doi: 10.1016/j.intimp.2025.114430, 40101415

[27] Ikeda Y Hamano H Horinouchi Y Miyamoto L Hirayama T Nagasawa H . Role of ferroptosis in cisplatin-induced acute nephrotoxicity in mice. J Trace Elem Med Biol. (2021) 67:126798. doi: 10.1016/j.jtemb.2021.126798, 34087581

[28] Song J Sheng J Lei J Gan W Yang Y. Mitochondrial targeted antioxidant SKQ1 ameliorates acute kidney injury by inhibiting Ferroptosis. Oxidative Med Cell Longev. (2022) 2022:2223957. doi: 10.1155/2022/2223957, 36193064 PMC9526623

[29] Tian R Tang S Zhao J Hao Y Zhao L Han X . β-Hydroxybutyrate protects against cisplatin-induced renal damage via regulating Ferroptosis. Ren Fail. (2024) 46:2354918. doi: 10.1080/0886022X.2024.2354918, 38757723 PMC11104694

[30] Tao Y Fu S Lu J Fu B Liu S Li L. Salvianolic acid B attenuates Ferroptosis in acute kidney injury by targeting PRDX5. FASEB J. (2025) 39:e70803. doi: 10.1096/fj.202500258RR, 40654183 PMC12257431

[31] Dong XQ Chu LK Cao X Xiong QW Mao YM Chen CH . Glutathione metabolism rewiring protects renal tubule cells against cisplatin-induced apoptosis and ferroptosis. Redox Rep. (2023) 28:2152607. doi: 10.1080/13510002.2022.2152607, 36692085 PMC9879199

[32] Li D Xie X Zhan Z Li N Yin N Yang S . HIF-1 induced tiRNA-Lys-CTT-003 is protective against cisplatin induced ferroptosis of renal tubular cells in mouse AKI model. Biochim Biophys Acta Mol basis Dis. (2024) 1870:167277. doi: 10.1016/j.bbadis.2024.167277, 38871033

[33] Zhang Y Hu J Zhang Y Ci X. Amentoflavone protects against cisplatin-induced acute kidney injury by modulating Nrf2-mediated oxidative stress and ferroptosis and partially by activating Nrf2-dependent PANoptosis. Front Pharmacol. (2025) 16:1508047. doi: 10.3389/fphar.2025.1508047 40110131 PMC11919867

[34] Lai K Chen Z Lin S Ye K Yuan Y Li G . The IDH1-R132H mutation aggravates cisplatin-induced acute kidney injury by promoting ferroptosis through disrupting NDUFA1 and FSP1 interaction. Cell Death Differ. (2025) 32:242–55. doi: 10.1038/s41418-024-01381-8, 39306640 PMC11802792

[35] Yuksel B Ozkan AD Aydın D Betts Z. Evaluation of the antioxidative and genotoxic effects of sodium butyrate on breast cancer cells. Saudi J Biol Sci. (2022) 29:1394–401. doi: 10.1016/j.sjbs.2021.12.061, 35280546 PMC8913555

[36] Ozkan AD Betts Z Yuksel B Alimudin J Aydin D Guzel E. Assessing in vitro anti-cancer efficacy of sulfonated water-soluble phthalocyanine on breast and prostate cancer cells. J Porphyrins Phthalocyanines. (2023) 27:493–500. doi: 10.1142/S1088424623500256

