# Supplementary material for: Ferroptosis inhibition as a renoprotective strategy in cisplatin-induced acute kidney injury: multilevel meta-analysis of mechanistic biomarkers
Source: Front Med (Lausanne). 2026 May 4;13:1801504. doi: 10.3389/fmed.2026.1801504 (PMC13180951; doi:10.3389/fmed.2026.1801504)
Supplement: Supplementary file 1 [file Supplementary_file_1.DOCX]

Appendix

Abdel-Rahman, N., Abdel-Rahman, A. M., & Sharawy, M. H. (2025). Regulating ferroptosis by roflumilast attenuates cisplatin-induced kidney injury. *International Immunopharmacology*, *151*, 114331. doi.org/10.1016/j.intimp.2025.114331

Airik, M., Clayton, K., Wipf, P., & Airik, R. (2024). JP4-039 Mitigates Cisplatin-Induced Acute Kidney Injury by Inhibiting Oxidative Stress and Blocking Apoptosis and Ferroptosis in Mice. *Antioxidants*, *13*(12), 1534. doi.org/10.3390/ antiox13121534

Dai, Q., Xiang, Y., Qiang, R., Li, G., Song, Y., Yu, Y., ... & Liu, X. (2025). Aloe-emodin mitigates cisplatin-induced acute kidney injury by Nrf2-mediated ferroptosis regulation. *Free Radical Biology and Medicine*. doi.org/10.1016/j.freeradbiomed.2025.09.016

Dong, X. Q., Chu, L. K., Cao, X., Xiong, Q. W., Mao, Y. M., Chen, C. H., ... & Yan, X. M. (2023). Glutathione metabolism rewiring protects renal tubule cells against cisplatin-induced apoptosis and ferroptosis. *Redox Report*, *28*(1), 2152607. doi.org/10.1080/13510002.2022.2152607

Cai, F., Li, D., Xie, Y., Wang, X., Ma, H., Xu, H., ... & Hua, Z. C. (2024). Sulfide: quinone oxidoreductase alleviates ferroptosis in acute kidney injury via ameliorating mitochondrial dysfunction of renal tubular epithelial cells. *Redox Biology*, *69*, 102973. doi.org/10.1016/j.redox.2023.102973

Cai, F., Li, D., Zhou, K., Zhang, W., & Yang, Y. (2024). Tiliroside attenuates acute kidney injury by inhibiting ferroptosis by disrupting the NRF2-KEAP1 interaction. *Phytomedicine*, *126*, 155407. doi.org/10.1016/j.phymed.2024.155407

Cao, Y., Liu, X., Guo, C., Yang, W., Wang, X., Wang, X., ... & Chen, D. (2025). Biomimetic reactive oxygen/nitrogen nanoscavengers inhibit “ferroptosis storm” and modulate immune targeting for acute kidney injury. *Journal of Controlled Release*, *379*, 59-76. doi.org/10.1016/j.jconrel.2025.01.006

Chen, H., Guo, R., Zhang, C., Zhang, L., Hu, H., Du, P., ... & Jiang, T. (2025). Mechanistic insights into the renoprotective effects of artesunate in cisplatin-induced acute kidney injury. *Life Sciences*, 123820 doi.org/10.1016/j.lfs.2025.123820

Chen, W., Wang, B., Liang, S., Zheng, L., Fang, H., Xu, S., ... & Feng, W. (2025). Fullerenols as efficient ferroptosis inhibitor by targeting lipid peroxidation for preventing drug-induced acute kidney injury. *Journal of Colloid and Interface Science*, *680*, 261-273. doi.org/10.1016/j.jcis.2024.10.198

Dong, X. Q., Chu, L. K., Cao, X., Xiong, Q. W., Mao, Y. M., Chen, C. H., ... & Yan, X. M. (2023). Glutathione metabolism rewiring protects renal tubule cells against cisplatin-induced apoptosis and ferroptosis. *Redox Report*, *28*(1), 2152607.

Guan, K., Zhang, Y., Guo, S., Ning, X., & Sun, S. (2025). DPP8 and DPP9 promote tubular epithelial cell ferroptosis in acute kidney injury. *European Journal of Medical Research*, *30*(1), 630. doi.org/10.1186/s40001-025-02861-4

Guo, S., Zhou, L., Liu, X., Gao, L., Li, Y., & Wu, Y. (2024). Baicalein alleviates cisplatin-induced acute kidney injury by inhibiting ALOX12-dependent ferroptosis. *Phytomedicine*, *130*, 155757. doi.org/10.1016/j.phymed.2024.155757

Hu, Z., Zhang, H., Yi, B., Yang, S., Liu, J., Hu, J., ... & Zhang, W. (2020). Nitrogen VDR activation attenuate cisplatin induced AKI by inhibiting ferroptosis. *Cell death & disease*, *11*(1), 73. doi.org/10.1038/s41419-020-2256-z

Hu, Z., Zhang, H., Yi, B., Yang, S., Liu, J., Hu, J., ... & Zhang, W. (2020). VDR activation attenuate cisplatin induced AKI by inhibiting ferroptosis. *Cell death & disease*, *11*(1), 73. doi.org/10.1038/s41419-020-2256-z

Hu, J., Gu, W., Ma, N., Fan, X., & Ci, X. (2022). Leonurine alleviates ferroptosis in cisplatin‐induced acute kidney injury by activating the Nrf2 signalling pathway. *British Journal of Pharmacology*, *179*(15), 3991-4009. DOI: 10.1111/bph.15834

Hu, J., Zhang, Y., Zhang, Y., Shi, N., Miu, Y., Huang, J., ... & Ci, X. (2025). Bergenin inhibits ferritinophagy and ferroptosis in cisplatin-induced acute kidney injury by activating the p-GSK3β/Nrf2/PPARγ pathway. *International Immunopharmacology*, *147*, 114004. doi.org/10.1016/j.intimp.2024.114004

Ikeda, Y., Hamano, H., Horinouchi, Y., Miyamoto, L., Hirayama, T., Nagasawa, H., ... & Tsuchiya, K. (2021). Role of ferroptosis in cisplatin-induced acute nephrotoxicity in mice. *Journal of Trace Elements in Medicine and Biology*, *67*, 126798. doi.org/10.1016/j.jtemb.2021.126798

Ji, Y., Du, S., Li, J., Ma, H., Wang, X., Hao, Y., ... & He, S. (2025). Discovery of Zharp1-163 as a dual inhibitor of ferroptosis and necroptosis for the treatment of inflammatory disorders and kidney injury. *Cell Death Discovery*, *11*(1), 413. doi.org/10.1038/s41420-025-02693-5

Jin, X., He, R., Lin, Y., Liu, J., Wang, Y., Li, Z., ... & Yang, S. (2023). Shenshuaifu granule attenuates acute kidney injury by inhibiting ferroptosis mediated by p53/SLC7A11/GPX4 pathway. *Drug Design, Development and Therapy*, 3363-3383. doi.org/10.2147/DDDT.S433994

Jiao, N., Wang, B., Zhang, Q., Liu, Y., & Zhao, F. (2025). 4-Methoxylonchocarpin protects against cisplatin induced acute kidney injury via regulating ferroptosis. *Renal Failure*, *47*(1), 2545941. doi.org/10.1080/0886022X.2025.2545941

Kim, D. U., Kim, D. G., Choi, J. W., Shin, J. Y., Kweon, B., Zhou, Z., ... & Park, S. J. (2021). Loganin attenuates the severity of acute kidney injury induced by cisplatin through the inhibition of ERK activation in mice. *International Journal of Molecular Sciences*, *22*(3), 1421.

Kim, D. H., Choi, H. I., Park, J. S., Kim, C. S., Bae, E. H., Ma, S. K., & Kim, S. W. (2022). Farnesoid X receptor protects against cisplatin-induced acute kidney injury by regulating the transcription of ferroptosis-related genes. *Redox Biology*, *54*, 102382. doi.org/10.1016/j.redox.2022.102382

Lai, K., Chen, Z., Lin, S., Ye, K., Yuan, Y., Li, G., ... & Xu, Y. (2025). The IDH1-R132H mutation aggravates cisplatin-induced acute kidney injury by promoting ferroptosis through disrupting NDUFA1 and FSP1 interaction. *Cell Death & Differentiation*, *32*(2), 242-255. doi.org/10.1038/s41418-024-01381-8

Li, Y., Li, K., Zhao, W., Wang, H., Xue, X., Chen, X., ... & Fu, R. (2023). VPA improves ferroptosis in tubular epithelial cells after cisplatin-induced acute kidney injury. *Frontiers in Pharmacology*, *14*, 1147772 doi: 10.3389/fphar.2023.1147772

Li, D., Xie, X., Zhan, Z., Li, N., Yin, N., Yang, S., ... & Zhang, W. (2024). HIF-1 induced tiRNA-Lys-CTT-003 is protective against cisplatin induced ferroptosis of renal tubular cells in mouse AKI model. *Biochimica et Biophysica Acta (BBA)-Molecular Basis of Disease*, *1870*(7), 167277. doi.org/10.1016/j.bbadis.2024.167277

Li, J., Fu, C., Feng, B., Liu, Q., Gu, J., Khan, M. N., ... & Wu, H. (2024). Polyacrylic acid‐coated selenium‐doped carbon dots inhibit ferroptosis to alleviate chemotherapy‐associated acute kidney injury. *Advanced Science*, *11*(28), 2400527. DOI:10.1002/advs.202400527

Li, H., Xu, K., Mao, W., Yu, B., Liu, Z., Huang, F., & Yang, Z. (2025). Morroniside alleviates cisplatin-induced renal injury and gut dysbiosis via the gut–kidney axis and ferroptosis. *International Immunopharmacology*, *153*, 114430. doi.org/10.1016/j.intimp.2025.114430

Liang, N. N., Guo, Y. Y., Zhang, X. Y., Ren, Y. H., He, Y. Z., Liu, Z. B., ... & Xu, S. (2024). Mitochondrial Dysfunction‐Evoked DHODH Acetylation is Involved in Renal Cell Ferroptosis during Cisplatin‐Induced Acute Kidney Injury. *Advanced Science*, *11*(43), 2404753. doi.org/10.1002/advs.202404753

Meng, X., Huang, W., Mo, W., Shu, T., Yang, H., & Ning, H. (2021). ADAMTS-13-regulated nuclear factor E2-related factor 2 signaling inhibits ferroptosis to ameliorate cisplatin-induced acute kidney injuy: Running title: Role of ADAMTS-13 and ferroptosis in AKI. *Bioengineered*, *12*(2), 11610-11621. doi.org/10.1080/21655979.2021.1994707

Mishima, E., Sato, E., Ito, J., Yamada, K. I., Suzuki, C., Oikawa, Y., ... & Abe, T. (2020). Drugs repurposed as antiferroptosis agents suppress organ damage, including AKI, by functioning as lipid peroxyl radical scavengers. *Journal of the American Society of Nephrology*, *31*(2), 280-296. doi.org/10.1681/ASN.2019060570

Pan, M., Wang, Z., Wang, Y., Jiang, X., Fan, Y., Gong, F., ... & Wang, D. (2023). Celastrol alleviated acute kidney injury by inhibition of ferroptosis through Nrf2/GPX4 pathway. *Biomedicine & Pharmacotherapy*, *166*, 115333. doi.org/10.1016/j.biopha.2023.115333

Razek, N. S. A., Nassar, N. N., Sayed, R. H., El-Sahar, A. E., & Abdallah, D. M. (2025). Liraglutide orchestrates ferroptosis defense against murine cisplatin acute kidney injury: NRF2 activation via both KEAP1-dependent and-independent mechanisms is essential for SLC7A11/GPX4 renoprotection. *Journal of Trace Elements in Medicine and Biology*, 127755. doi.org/10.1016/j.jtemb.2025.127755

Sharawy, N., Aboulhoda, B. E., Khalifa, M. M., Morcos, G. N., Morsy, S. A. A. G., Alghamdi, M. A., ... & Abd Algaleel, W. A. (2024). Amelioration of nephrotoxicity by targeting ferroptosis: role of NCOA4, IREB2, and SLC7a11 signaling. *Brazilian Journal of Medical and Biological Research*, *57*, e13116. doi: 10.1590/1414-431X2024e13116

Shi, M., Mobet, Y., & Shen, H. (2024). Quercetin attenuates acute kidney injury caused by cisplatin by inhibiting ferroptosis and cuproptosis. *Cell Biochemistry and Biophysics*, *82*(3), 2687-2699. doi.org/10.1007/s12013-024-01379-6

Song, J., Sheng, J., Lei, J., Gan, W., & Yang, Y. (2022). Mitochondrial targeted antioxidant SKQ1 ameliorates acute kidney injury by inhibiting ferroptosis. *Oxidative Medicine and Cellular Longevity*, *2022*(1), 2223957. doi.org/10.1155/2022/2223957

Song, Z., Li, Z., Pan, T., Liu, T., Gong, B., Wang, Z., ... & Fan, H. (2024). Protopanaxadiol prevents cisplatin-induced acute kidney injury by regulating ferroptosis. *Journal of Pharmacy and Pharmacology*, *76*(7), 884-896. doi.org/10.1093/jpp/rgae050

Sun, M., Chang, H., Jiang, F., Zhang, W., Yang, Q., Wang, X., ... & Wang, Y. (2024). Hazel leaf polyphenol extract alleviated cisplatin-induced acute kidney injury by reducing ferroptosis through inhibiting Hippo signaling. *Molecules*, *29*(8), 1729 doi.org/10.3390/molecules29081729

Tang, Z., Chen, K., Sun, C., Ying, X., & Li, M. (2024). Cordycepin inhibits kidney injury by regulating GSK‐3β‐mediated Nrf2 activation. *Journal of Biochemical and Molecular Toxicology*, *38*(1), e23600. doi.org/10.1002/jbt.23600

Tao, Y., Fu, S., Lu, J., Fu, B., Liu, S., & Li, L. (2025). Salvianolic Acid B Attenuates Ferroptosis in Acute Kidney Injury by Targeting PRDX5. *The FASEB Journal*, *39*(14), e70803. doi.org/10.1096/fj.202500258RR

Tian, M., Wang, L., Dong, Z., Wang, X., Qin, X., Wang, C., ... & Huang, Q. (2022). Preparation, structural characterization, antioxidant activity and protection against cisplatin-induced acute kidney injury by polysaccharides from the lateral root of Aconitum carmichaelii. *Frontiers in pharmacology*, *13*, 1002774. doi: 10.3389/fphar.2022.1002774

Tian, R., Tang, S., Zhao, J., Hao, Y., Zhao, L., Han, X., ... & Zhou, X. (2024). β-Hydroxybutyrate protects against cisplatin-induced renal damage via regulating ferroptosis. *Renal Failure*, *46*(1), 2354918. doi.org/10.1080/0886022X.2024.2354918

Tu, Y., Tang, E., Ye, H., Xiang, Q., Ye, Z., Hao, Y., & Liao, W. (2025). Flammulina Velutipes polysaccharides ameliorate cisplatin-induced acute kidney injury in mice via regulation of gut microbiota and Ferroptosis pathway. *International Journal of Biological Macromolecules*, *290*, 138526. doi.org/10.1016/j.ijbiomac.2024.138526

Qi, H., Deng, F., Wang, Y., Zhang, H., Kanwar, Y. S., & Dai, Y. (2022). Myo-inositol supplementation alleviates cisplatin-induced acute kidney injury via inhibition of ferroptosis. *Cells*, *12*(1), 16. doi.org/10.3390/ cells12010016

Qiu, C. W., Chen, B., Zhu, H. F., Liang, Y. L., & Mao, L. S. (2024). Gastrodin alleviates cisplatin nephrotoxicity by inhibiting ferroptosis via the SIRT1/FOXO3A/GPX4 signaling pathway. *Journal of Ethnopharmacology*, *319*, 117282. doi.org/10.1016/j.jep.2023.117282

Wang, S., Zheng, Y., Jin, S., Fu, Y., & Liu, Y. (2022). Dioscin protects against cisplatin-induced acute kidney injury by reducing ferroptosis and apoptosis through activating Nrf2/HO-1 signaling. *Antioxidants*, *11*(12), 2443. doi.org/ 10.3390/antiox11122443

Xu, Z., Zhang, M., Wang, W., Zhou, S., Yu, M., Qiu, X., ... & Zhang, G. (2023). Dihydromyricetin attenuates cisplatin-induced acute kidney injury by reducing oxidative stress, inflammation and ferroptosis. *Toxicology and Applied Pharmacology*, *473*, 116595. doi.org/10.1016/j.taap.2023.116595

Yuan, X., Long, L., Wang, M., Chen, W., Liang, B., Xu, L., ... & Li, C. (2025). Rhein Alleviates Cisplatin-Induced Acute Kidney Injury via Downregulation of NOX4-COX2/PGFS Signaling Pathway. *Drug Design, Development and Therapy*, 4641-4664.

Zeng, F., Qin, Y., Nijiati, S., Liu, Y., Ye, J., Shen, H., ... & Zhou, Z. (2024). Ultrasmall Nanodots with Dual Anti‐Ferropototic Effect for Acute Kidney Injury Therapy. *Advanced Science*, *11*(39), 2403305. DOI:10.1002/advs.202403305

Zhang, Z., Zhou, H., Gu, W., Wei, Y., Mou, S., Wang, Y., ... & Zhong, Q. (2024). CGI1746 targets σ1R to modulate ferroptosis through mitochondria-associated membranes. *Nature Chemical Biology*, *20*(6), 699-709. doi.org/10.1038/s41589-023-01512-1

Zhang, Y., Hu, J., Zhang, Y., & Ci, X. (2025). Amentoflavone protects against cisplatin-induced acute kidney injury by modulating Nrf2-mediated oxidative stress and ferroptosis and partially by activating Nrf2-dependent PANoptosis. *Frontiers in Pharmacology*, *16*, 1508047

Zhao, M., Lin, Y., Zeng, Q., Li, Y., Feng, J., Chen, X., ... & Li, L. (2025). Entospletinib alleviates acute liver injury and acute kidney injury by inhibiting ferroptosis. *European Journal of Pharmacology*, 177939. doi.org/10.1016/j.ejphar.2025.177939

Zheng, D., Ruan, X., Wu, Q., Qiu, Y., & Ruan, S. (2024). Yishen Jiangzhuo decoction attenuates cisplatin‑induced acute kidney injury by inhibiting inflammation, oxidative stress and apoptosis through the TNF signal pathway. *Experimental and Therapeutic Medicine*, *28*(2), 331. DOI: 10.3892/etm.2024.12620

Zhong, D., Quan, L., Hao, C., Chen, J., Qiao, R., Lin, T., ... & Sun, Y. (2023). Targeting mPGES-2 to protect against acute kidney injury via inhibition of ferroptosis dependent on p53. *Cell Death & Disease*, *14*(10), 710.  doi.org/10.1038/s41419-023-06236-7

Zhou, L., Yu, P., Wang, T. T., Du, Y. W., Chen, Y., Li, Z., ... & Liu, H. B. (2022). Polydatin attenuates cisplatin‐induced acute kidney injury by inhibiting ferroptosis. *Oxidative medicine and cellular longevity*, *2022*(1), 9947191. doi.org/10.1155/2022/9947191

Zhu, Z., Liu, X., Li, P., Wang, H., Zhang, Y., Liu, M., & Ren, J. (2023). Renal clearable quantum dot–drug conjugates modulate labile iron species and scavenge free radicals for attenuating chemotherapeutic drug-induced acute kidney injury. *ACS Applied Materials & Interfaces*, *15*(18), 21854-21865. doi.org/10.1021/acsami.3c00714

Zhu, J., Yuan, A., Le, Y., Chen, X., Guo, J., Liu, J., ... & Lu, K. (2024). Yi-Qi-Jian-Pi-Xiao-Yu formula inhibits cisplatin-induced acute kidney injury through suppressing ferroptosis via STING-NCOA4-mediated ferritinophagy. *Phytomedicine*, *135*, 156189. doi.org/10.1016/j.phymed.2024.156189

Zhu, H., Wu, X., Tan, Y., Shi, L., Bai, W., & Li, X. (2025). Anthocyanin-functionalized selenocysteine nanotherapeutics alleviate cisplatin nephrotoxicity by inhibiting oxidative stress and ferroptosis. *Journal of Nanobiotechnology*, *23*(1), 703. doi.org/10.1186/s12951-025-03741-z
