# Supplementary material for: Ferroptosis inhibition as a renoprotective strategy in cisplatin-induced acute kidney injury: multilevel meta-analysis of mechanistic biomarkers
Source: Front Med (Lausanne). 2026 May 4;13:1801504. doi: 10.3389/fmed.2026.1801504 (PMC13180951; doi:10.3389/fmed.2026.1801504)
Supplement: Supplementary file 2 [file Supplementary_file_2.docx]

Table S2. Characteristics of Included Studies

| **Study ID** | **Country** | **Animal Species** | **Animal Strain** | **Sex** | **Cisplatin Dose mg/kg** | **Treatment Name** | **Treatment Dose mg/kg** | **Duration** |
| --- | --- | --- | --- | --- | --- | --- | --- | --- |
| Guo et al., 2024 | China | Mice | C57BL/6J | Male | 20 | Baicalein | 100 | 3 Days |
| Kim et al., 2022 | South Korea | Mice | C57BL/6J | Male | 20 | GW4064 | 30 | 2 Days and 3 Days |
| Hu et al., 2020 | China | Mice | C57BL/6 | Male | 20 | Paricalcitol | 20 | 2 Days and 3 Days |
| Zhang et al., 2024 | China | Mice | C57BL/6 | Male | 15 | CGI1746 | 200 | 4 Days |
| Pan et al., 2023 | China | Mice | C57BL/6 | Male | 20 | Celastrol | 1 | 3 Days |
| Zhu et al., 2024 | China | Mice | C57BL/6 | Male | 20 | YQJPXY Herbal Formula | 30 | 2 Days |
| Liang et al., 2024 | China | Mice | C57BL/6 | Male | 20 | MitoQ | 5 | 3 Days |
| Lai et al., 2025 | China | Mice | C57BL/6 | Male | 10 | Liproxstatin-1 | 10 | 3 Days |
| Xu et al., 2023 | China | Mice | C57BL/6 | Male | 22 | Dihydromyricetin DHM | 500 | 3 Days |
| Shi et al., 2024 | China | Mice | C57BL/6 | Male | 20 | Quercetin | 100 | 2 Days |
| Xu et al., 2024 | China | Mice | C57BL/6 | Male | 20 | Reserpin | 5 | 3 Days |
| Qi et al., 2023 | China | Mice | C57BL/6 | Male | 20 | Myo-Inositol MI | 500 | 3 Days |
| Mishima et al., 2019 | Japan | Mice | C57BL/6N | Male | 16 | Promethazine PMZ | 20 | 4 Days |
| Airik et al., 2024 | USA | Mice | 129Sv-Elite | Male | 20 | JP4-039 | 10 | 3 Days |
| Wang et al., 2022 | China | Rats | Wistar | Male | 20 | Dioscin | 80 | 3 Days |
| Dong et al., 2023 | China | Mice | C57BL/6J | Male | 20 | Baicalein | 100 | 3 Days |
| Meng et al., 2021 | China | Mice | C57BL/6 | Male | 20 | ADAMTS-13 | 0.057 | 3 Days |
| Zhu et al., 2023 | China | Mice | C57BL/6 | Male | 15 | p-CDs-DFO QDCs | 10 | 3 Days |
| Song et al., 2024 | China | Mice | C57BL/6 | Male | 20 | Protopanaxadiol PPD | 80 | 3 Days |
| Cai et al., 2024 | China | Mice | C57BL/6 | Male | 25 | ADT-OH | 37 | 4 Days |
| Sun et al., 2024 | China | Mice | C57BL/6 | Male | 20 | Hazel Leaf Extract ZP | 500 | 2 Days |
| Tang et al., 2024 | China | Mice | C57BL/6 | Male | 5 | Cordycepin COR | 200 | 14 Days |
| Tian et al., 2022 | China | Mice | C57BL/6 | Male | 20 | RFP Polysaccharide | 800 | 4 Days |
| Li et al., 2023 | USA | Mice | C57BL/6 | Male | 20 | Valproic Acid VPA | 200 | 3 Days |
| Zhang et al., 2025 | China | Mice | C57BL/6 | Male | 20 | Amentoflavone AME | 10 | 3 Days |
| Kim et al., 2021 | Korea | Mice | C57BL/6 | Male | 10 | Loganin | 20 | 3 Days |
| Tian et al., 2024 | China | Mice | C57BL/6 | Male | 20 | β-hydroxybutyrate β-HB | 300 | 3 Days |
| Jiao et al., 2024 | China | Mice | C57BL/6 | Male | 20 | 4-Methoxylonchocarpin | 200 | 3 Days |
| Song et al., 2022 | China | Mice | C57BL/6 | Male | 20 | Visomitin SKQ1 | 0.2 | 3 Days |
| Zhou et al., 2022 | China | Mice | C57BL/6 | Male | 20 | Polydatin PD | 40 | 2 Days |
| Razek et al., 2025 | Egypt | Rats | Wistar | Male | 8 | liraglutide | 0.4 | 5 Days |
| Zeng et al., 2024 | China | Mice | C57BL/6 | Male | 10 | FerroD | 15 | 4 Days |
| Li et al., 2024 | China | Mice | C57BL/6 | Male | 20 | SeCD | 0.5 | 3 Days |
| Ji et al., 2025 | China | Mice | C57BL/6 | Male | 20 | Zharp1-163 | 5 | 3 Days |
| Zhong et al., 2023 | China | Mice | C57BL/6 | Male | 20 | SZ0232 | 1 | 7 Days |
| Guan et al., 2025 | China | Mice | C57BL/6 | Male | 20 | TC-E5007 | 10 | 3 Days |
| Zhu et al., 2025 | China | Mice | C57BL/6 | Male | 15 | Cyanidin-3-O-glucoside | 2 | 5 Days |
| Sharawy et al., 2024 | Egypt | Rats | sprague-Daw- | Male | 2 | Deferiprone | 200 | 10 Days |
| Li et al., 2025 | China | Mice | C57BL/6 | Male | 7 | Morroniside | 20 | 4 Days |
| Abdel-Rahman et al., 2025 | Egypt | Rats | Wistar | Male | 7 | Roflumilast | 3 | 10 Days |
| Hu et al., 2025 | China | Mice | C57BL/6 | Male | 20 | Bergenin | 40 | 3 Days |
| Cai et al., 2024 | China | Mice | C57BL/6 | Male | 6 | Tiliroside | 30 | 3 Days |
| Li et al., 2024 | China | Mice | C57BL/6 | Male | 20 | tiRNA-Lys-CTT-003 Mimic | 5 | 2 Days |
| Dai et al., 2025 | China | Mice | C57BL/6 | Male | 15 | Aloe-emodin - AE | 80 | 3 Days |
| Qiu et al., 2024 | China | Mice | C57BL/6 | Male | 20 | Gastrodin | 100 | 3 Days |
| Cao et al., 2025 | China | Mice | C57BL/6 | Male | 20 | M2FPPF@Cur | 5 | 3 Days |
| Tu et al., 2025 | China | Mice | C57BL/6 | Male | 30 | FVPs | 200 | 14 Days |
| Chen et al., 2025 | China | Mice | C57BL/6 | Male | 20 | Artesunate - ART | 300 | 9 Days |
| Chen et al., 2025 | China | Mice | C57BL/6 | Male | 20 | Fullerenol | 20 | 3 Days |
| Zhao et al., 2025 | China | Mice | C57BL/6 | Male | 20 | Entospletinib | 20 | 3 Days |
| Ikeda et al., 2021 | Japan | Mice | C57BL/6 | Male | 20 | Ferrostatin-1 | 5 | 3 Days |
| Hu et al., 2021 | China | Mice | C57BL/6 | Female | 20 | Leonurine | 100 | 3 Days |
| Zheng et al., 2024 | China | Mice | ICR | Male | 18.75 | Yishen Jiangzhuo | 14000 | 4 Days |
| Hu et al., 2020 | China | Mice | C57BL/6 | Male | 20 | Vitamin D Reseptör | 0.0002 | 7 Days |
| Li et al., 2024 | China | Mice | C57BL/6 | Male | 20 | Selenium-Doped Carbon Dots | 0.5 | 3 Days |
| Jin et al., 2023 | China | Mice | C57BL/6 | Male | 20 | Shenshuaifu granule l | 5200 | 3 Days |
| Yuan et al., 2025 | China | Mice | C57BL/6 | Male | 20 | Rhein | 80 | 3 Days |
| Tao et al., 2025 | China | Mice | C57BL/6 | Male | 20 | Salvianolic Acid B | 50 | 3 Days |
