# Supplementary material for: Ferroptosis inhibition as a renoprotective strategy in cisplatin-induced acute kidney injury: multilevel meta-analysis of mechanistic biomarkers
Source: Front Med (Lausanne). 2026 May 4;13:1801504. doi: 10.3389/fmed.2026.1801504 (PMC13180951; doi:10.3389/fmed.2026.1801504)
Supplement: Supplementary file 3 [file Supplementary_file_3.docx]

Supplementary Table S3 detailing the 5-domain Risk of Bias assessment for all 58 studies

| **Author (Year)** | **Selection Bias (Randomization)** | **Performance Bias (Standardization)** | **Detection Bias (Blinding)** | **Attrition Bias (Incomplete Data)** | **Reporting Bias (SD/N Reporting)** | **Overall Quality** |
| --- | --- | --- | --- | --- | --- | --- |
| Guo et al., 2024 | Unclear | Low Risk | Unclear | Low Risk | Low Risk | High |
| Kim et al., 2022 | Low Risk | Low Risk | Unclear | Low Risk | Low Risk | High |
| Hu et al., 2020 | Unclear | Low Risk | Unclear | Low Risk | Low Risk | High |
| Zhang et al., 2024 | Unclear | Low Risk | Unclear | Low Risk | Low Risk | Moderate |
| Pan et al., 2023 | Unclear | Low Risk | Unclear | Low Risk | Low Risk | High |
| Zhu et al., 2024 | Low Risk | Low Risk | Unclear | Low Risk | Low Risk | High |
| Liang et al., 2024 | Unclear | Low Risk | Unclear | Low Risk | Low Risk | High |
| Lai et al., 2025 | Unclear | Low Risk | Unclear | Low Risk | Low Risk | High |
| Xu et al., 2023 | Unclear | Low Risk | Unclear | Low Risk | Low Risk | High |
| Shi et al., 2024 | Unclear | Low Risk | Unclear | Low Risk | Low Risk | High |
| Xu et al., 2024 | Unclear | Low Risk | Unclear | Low Risk | Low Risk | Moderate |
| Qi et al., 2023 | Low Risk | Low Risk | Unclear | Low Risk | Low Risk | High |
| Mishima et al., 2019 | Unclear | Low Risk | Unclear | Low Risk | Low Risk | High |
| Airik et al., 2024 | Unclear | Low Risk | Unclear | Low Risk | Low Risk | High |
| Wang et al., 2022 | Unclear | Low Risk | Unclear | Low Risk | Low Risk | High |
| Dong et al., 2023 | Low Risk | Low Risk | Unclear | Low Risk | Low Risk | High |
| Meng et al., 2021 | Unclear | Low Risk | Unclear | Low Risk | Low Risk | High |
| Zhu et al., 2023 | Unclear | Low Risk | Unclear | Low Risk | Low Risk | Moderate |
| Song et al., 2024 | Unclear | Low Risk | Unclear | Low Risk | Low Risk | High |
| Cai et al., 2024 (ADT-OH) | Unclear | Low Risk | Unclear | Low Risk | Low Risk | High |
| Sun et al., 2024 | Low Risk | Low Risk | Unclear | Low Risk | Low Risk | High |
| Tang et al., 2024 | Unclear | Low Risk | Unclear | Low Risk | Low Risk | High |
| Tian et al., 2022 | Unclear | Low Risk | Unclear | Low Risk | Low Risk | High |
| Li et al., 2023 | Unclear | Low Risk | Unclear | Low Risk | Low Risk | High |
| Zhang et al., 2025 | Unclear | Low Risk | Unclear | Low Risk | Low Risk | Moderate |
| Kim et al., 2021 | Low Risk | Low Risk | Unclear | Low Risk | Low Risk | High |
| Tian et al., 2024 | Unclear | Low Risk | Unclear | Low Risk | Low Risk | High |
| Jiao et al., 2024 | Unclear | Low Risk | Unclear | Low Risk | Low Risk | High |
| Song et al., 2022 | Unclear | Low Risk | Unclear | Low Risk | Low Risk | High |
| Zhou et al., 2022 | Unclear | Low Risk | Unclear | Low Risk | Low Risk | High |
| Razek et al., 2025 | Low Risk | Low Risk | Unclear | Low Risk | Low Risk | High |
| Zeng et al., 2024 | Unclear | Low Risk | Unclear | Low Risk | Low Risk | High |
| Li et al., 2024 (SeCD) | Unclear | Low Risk | Unclear | Low Risk | Low Risk | Moderate |
| Ji et al., 2025 | Unclear | Low Risk | Unclear | Low Risk | Low Risk | High |
| Zhong et al., 2023 | Unclear | Low Risk | Unclear | Low Risk | Low Risk | High |
| Guan et al., 2025 | Low Risk | Low Risk | Unclear | Low Risk | Low Risk | High |
| Zhu et al., 2025 | Unclear | Low Risk | Unclear | Low Risk | Low Risk | High |
| Sharawy et al., 2024 | Unclear | Low Risk | Unclear | Low Risk | Low Risk | High |
| Li et al., 2025 | Unclear | Low Risk | Unclear | Low Risk | Low Risk | Moderate |
| Abdel-Rahman et al., 2025 | Unclear | Low Risk | Unclear | Low Risk | Low Risk | High |
| Hu et al., 2025 | Low Risk | Low Risk | Unclear | Low Risk | Low Risk | High |
| Cai et al., 2024 (Tiliroside) | Unclear | Low Risk | Unclear | Low Risk | Low Risk | High |
| Li et al., 2024 (tiRNA) | Unclear | Low Risk | Unclear | Low Risk | Low Risk | High |
| Dai et al., 2025 | Unclear | Low Risk | Unclear | Low Risk | Low Risk | High |
| Qiu et al., 2024 | Unclear | Low Risk | Unclear | Low Risk | Low Risk | Moderate |
| Cao et al., 2025 | Low Risk | Low Risk | Unclear | Low Risk | Low Risk | High |
| Tu et al., 2025 | Unclear | Low Risk | Unclear | Low Risk | Low Risk | High |
| Chen et al., 2025 (ART) | Unclear | Low Risk | Unclear | Low Risk | Low Risk | High |
| Chen et al., 2025 (Fullerenol) | Unclear | Low Risk | Unclear | Low Risk | Low Risk | High |
| Zhao et al., 2025 | Unclear | Low Risk | Unclear | Low Risk | Low Risk | High |
| Ikeda et al., 2021 | Low Risk | Low Risk | Unclear | Low Risk | Low Risk | High |
| Hu et al., 2021 | Unclear | Low Risk | Unclear | Low Risk | Low Risk | Moderate |
| Zheng et al., 2024 | Unclear | Low Risk | Unclear | Low Risk | Low Risk | High |
| Hu et al., 2020 (Vitamin D) | Unclear | Low Risk | Unclear | Low Risk | Low Risk | High |
| Li et al., 2024 (Selenium) | Unclear | Low Risk | Unclear | Low Risk | Low Risk | High |
| Jin et al., 2023 | Low Risk | Low Risk | Unclear | Low Risk | Low Risk | High |
| Yuan et al., 2025 | Unclear | Low Risk | Unclear | Low Risk | Low Risk | High |
| Tao et al., 2025 | Unclear | Low Risk | Unclear | Low Risk | Low Risk | High |
