# Supplementary material for: Ferroptosis inhibition as a renoprotective strategy in cisplatin-induced acute kidney injury: multilevel meta-analysis of mechanistic biomarkers
Source: Front Med (Lausanne). 2026 May 4;13:1801504. doi: 10.3389/fmed.2026.1801504 (PMC13180951; doi:10.3389/fmed.2026.1801504)

Funnel plot illustrating the treatment effects of GPX4 and GSH synthetic and natural compounds.
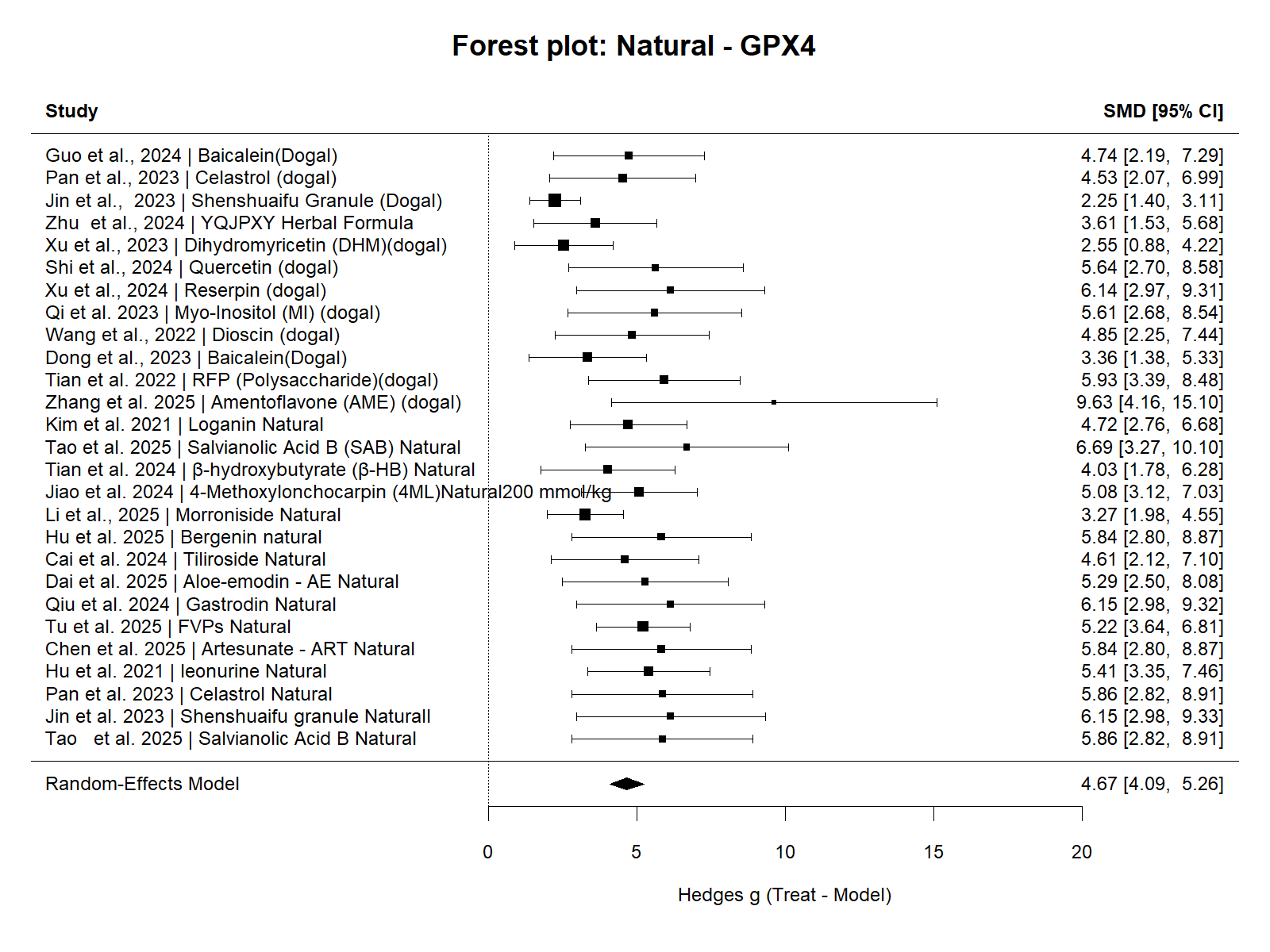


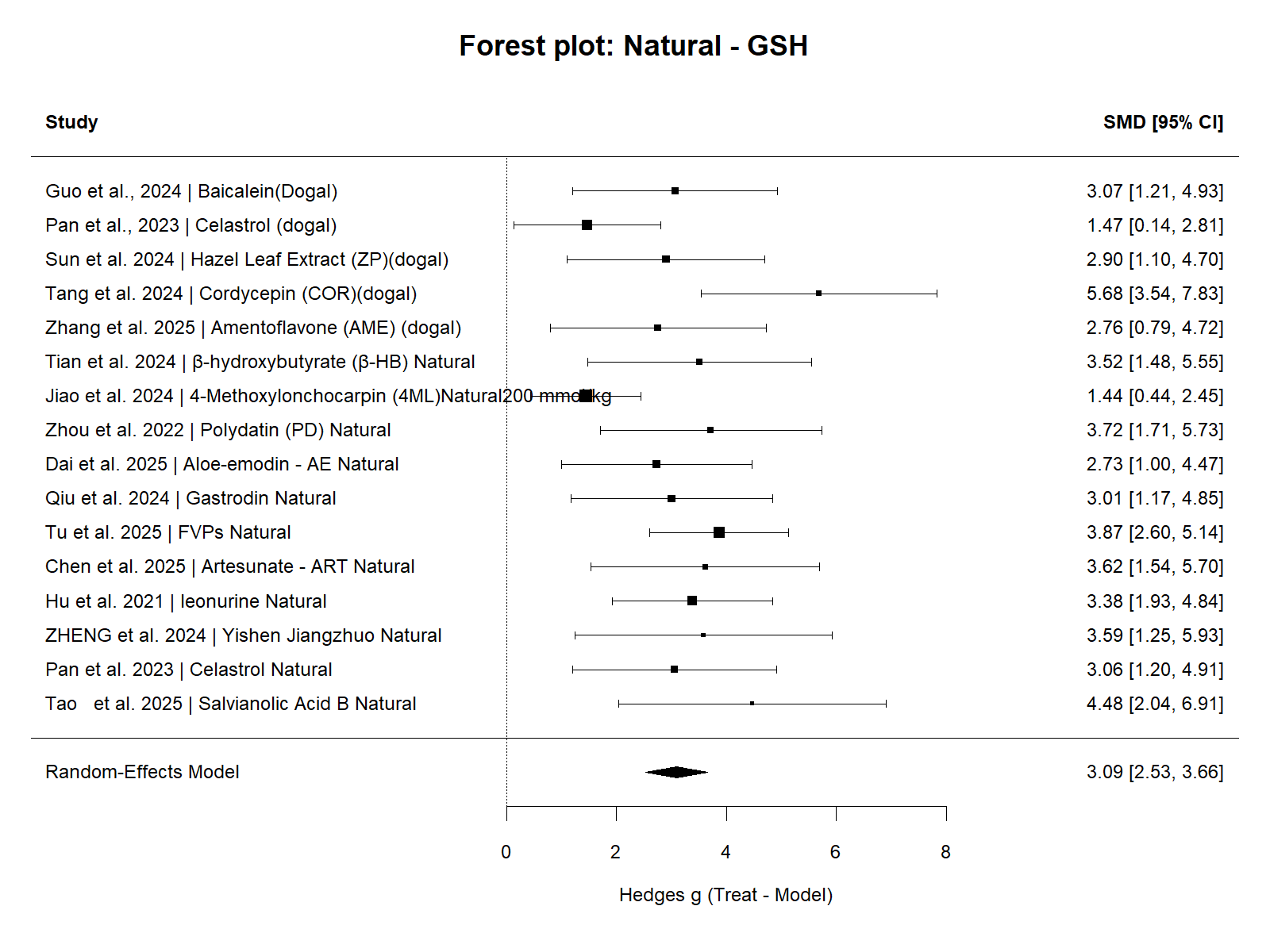


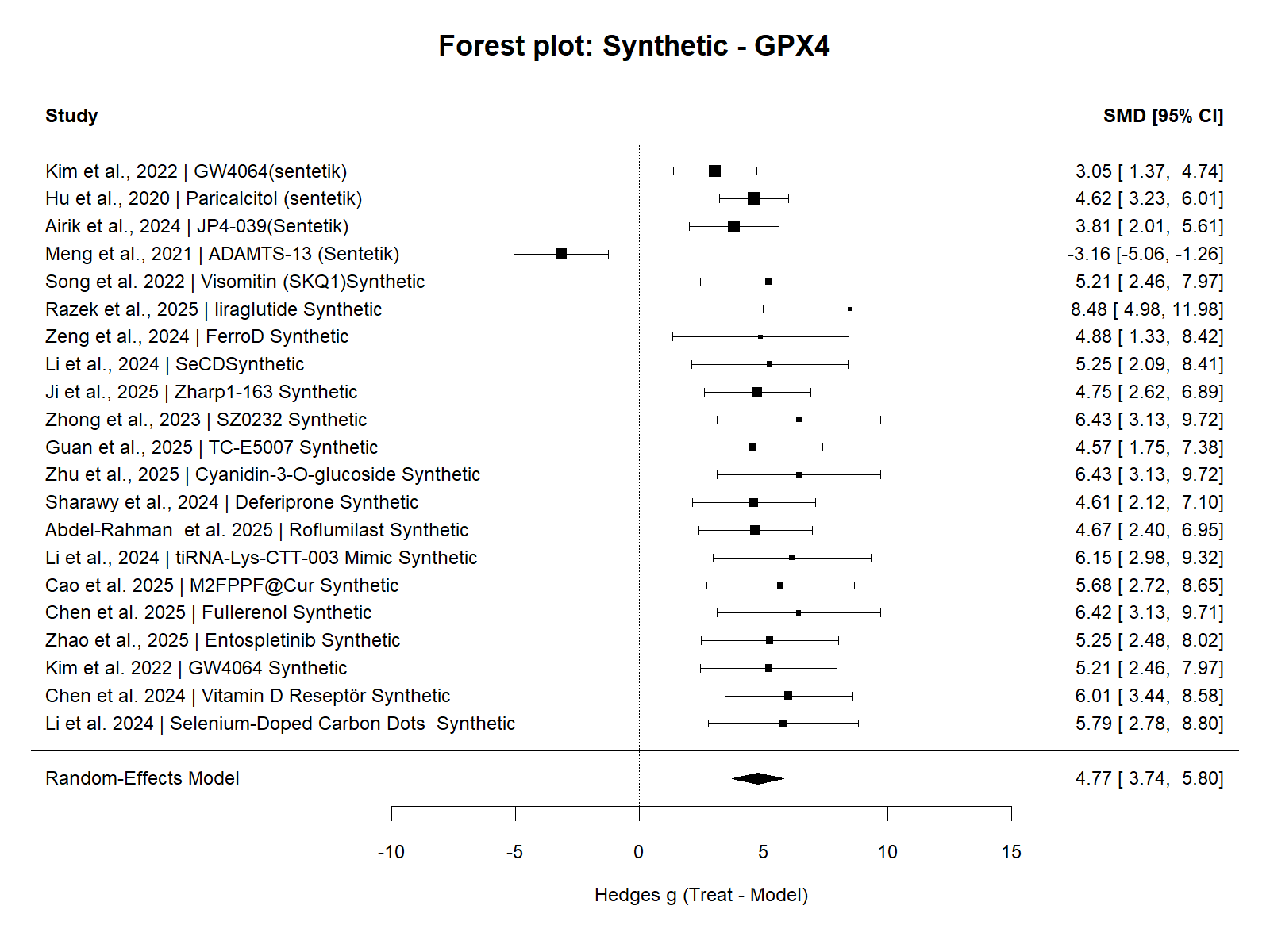


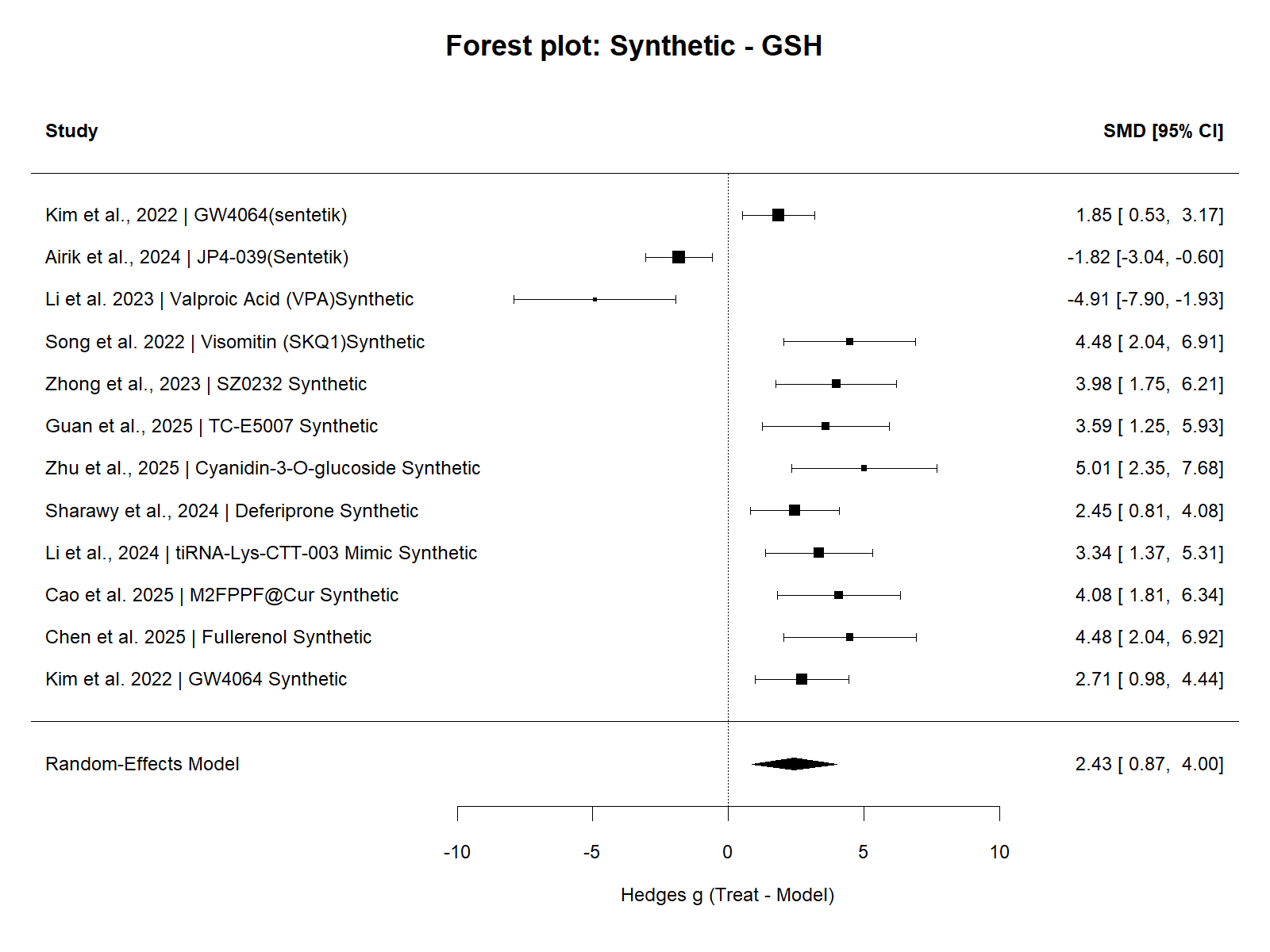

Supplement: Supplementary file 4 [file Supplementary_file_4.docx]
